# Non-native autoinducer analogs capable of modulating the SdiA quorum sensing receptor in *Salmonella enterica* serovar Typhimurium

**DOI:** 10.3762/bjoc.14.243

**Published:** 2018-10-17

**Authors:** Matthew J Styles, Helen E Blackwell

**Affiliations:** 1 Department of Chemistry, University of Wisconsin–Madison, 1101 University Avenue, Madison, WI 53706, USA

**Keywords:** *N*-acyl L-homoserine lactone, LuxR-type receptor, Quorum sensing, *Salmonella enterica* serovar Typhimurium, SdiA

## Abstract

Quorum sensing (QS) allows many common bacterial pathogens to coordinate group behaviors such as virulence factor production, host colonization, and biofilm formation at high population densities. This cell–cell signaling process is regulated by *N***-**acyl L-homoserine lactone (AHL) signals, or autoinducers, and LuxR-type receptors in Gram**-**negative bacteria. SdiA is an orphan LuxR-type receptor found in *Escherichia, Salmonella, Klebsiella, and Enterobacter* genera that responds to AHL signals produced by other species and regulates genes involved in several aspects of host colonization. The inhibition of QS using non-native small molecules that target LuxR-type receptors offers a non-biocidal approach for studying, and potentially controlling, virulence in these bacteria. To date, few studies have characterized the features of AHLs and other small molecules capable of SdiA agonism, and no SdiA antagonists have been reported. Herein, we report the screening of a set of AHL analogs to both uncover agonists and antagonists of SdiA and to start to delineate structure–activity relationships (SARs) for SdiA:AHL interactions. Using a cell-based reporter of SdiA in *Salmonella enterica* serovar Typhimurium, several non-natural SdiA agonists and the first set of SdiA antagonists were identified and characterized. These compounds represent new chemical probes for exploring the mechanisms by which SdiA functions during infection and its role in interspecies interactions. Moreover, as SdiA is highly stable when produced in vitro, these compounds could advance fundamental studies of LuxR-type receptor:ligand interactions that engender both agonism and antagonism.

## Introduction

In the fight against bacterial infections, microbes have a decisive advantage over the medical community: evolution [1]. Using bacteriocidal or bacteriostatic chemotherapies to treat infection imposes evolutionary pressures that drive rapid resistance development and spread within and among bacterial populations [2–3]. Antibiotic resistant clinical isolates have been observed for almost every known antibiotic, and the pace of new antibiotic discovery has lagged behind [4]. In recent years, non-bacteriocidal approaches have emerged as a new therapeutic strategy to treat infection with potentially a lesser propensity for resistance development and spread [4–6]. Interfering with the regulation of virulence phenotypes represents one such approach to complement antibiotics, and the interception of quorum sensing (QS) in bacteria has attracted considerable attention in this regard [7–9].

**Figure 1:**
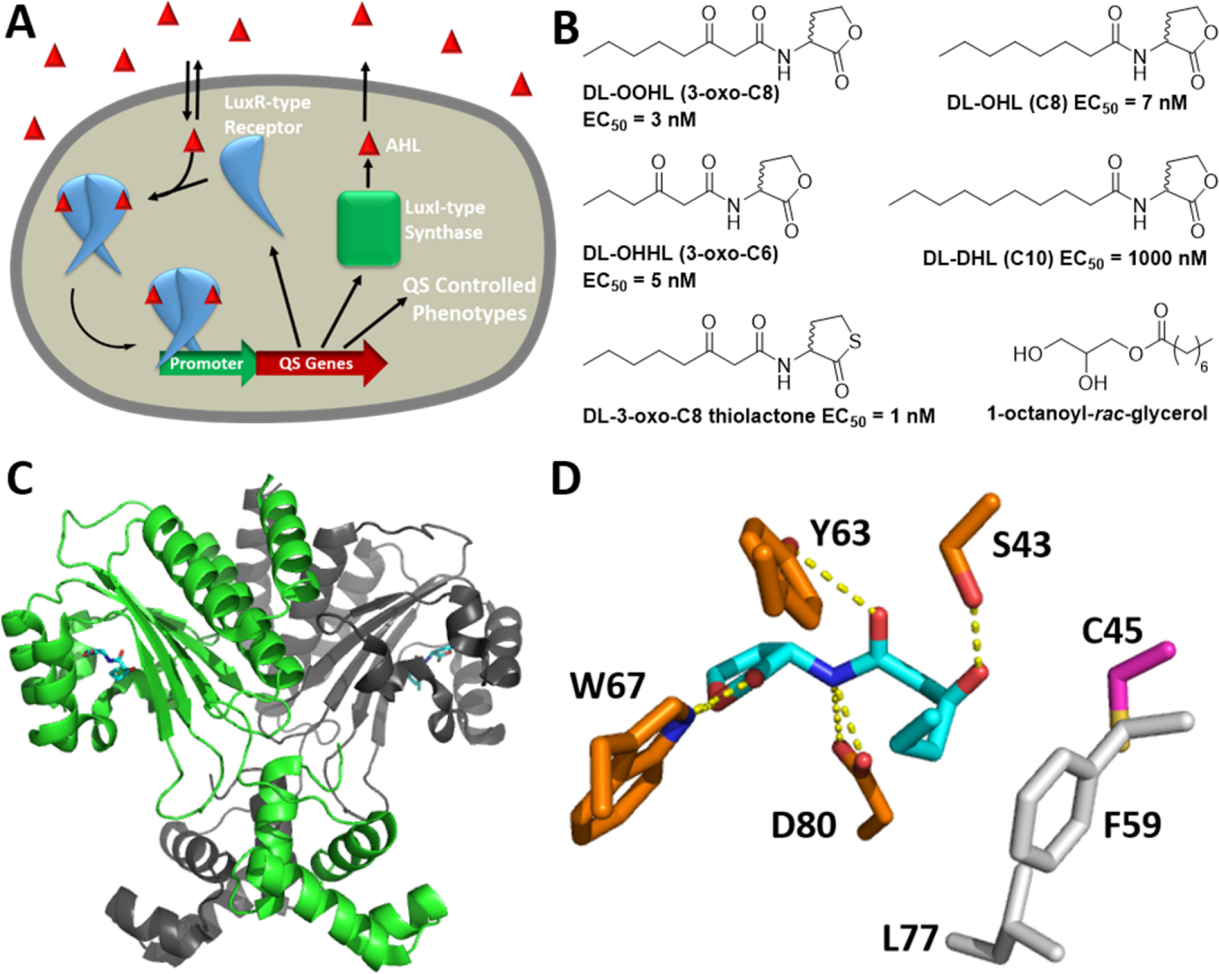
A) Overview of LuxI/LuxR-type QS. The LuxI-type protein produces the AHL signal. The AHL diffuses out of the cell and into the environment and other neighboring cells. At high population density, the intracellular concentration of AHL is sufficiently high to productively bind to the LuxR-type receptor protein. AHL binding typically promotes receptor homodimerization and binding to DNA at various promoters to activate transcription of QS-controlled genes, often including *luxI* and *luxR* (thereby autoinducing the QS system). B) Compounds previously reported to modulate SdiA (all examined as racemates) [20]. Their reported EC_50_ values in a *S.* Typhimurium SdiA reporter system are shown. L-OOHL (3-oxo-C8), L-OHHL (3-oxo-C6), L-OHL (C8), L-DHL (C10), and 1-octanoyl-*rac*-glycerol have been crystalized with SdiA from enterohemorrhagic *E. coli* (EHEC) [21–22]. C) The solid-state structure of the SdiA homodimer (PDB 4Y15; monomers shown in green and grey) bound to OOHL (cyan) [22]. D) Selected residues surrounding OOHL (cyan) in the SdiA ligand binding pocket (from PDB 4Y15 [22]): hydrogen bond acceptors/donors (orange), hydrophobic residues (grey), and a cysteine that potentially could be involved in inhibition (purple, see discussion below).

QS, a type of intra- and interspecies chemical communication, has been found to occur in many common bacterial pathogens [10–11]. These pathogens use QS to coordinate group beneficial behaviors such as virulence factor production, host colonization, and biofilm formation at high population densities [12]. Gram-negative bacteria typically use *N*-acyl L-homoserine lactone (AHL) signals for QS, which are produced by LuxI-type synthases and sensed by intracellular LuxR-type receptors (Figure 1A) [13]. The AHL signals are produced at a low, but constant basal level, and rapidly diffuse into the local environment. As the population grows, so does the concentration of AHL, and once it reaches a threshold intracellular level (and thus a “quorum” has been achieved), productive AHL:LuxR-type protein binding occurs that activates the transcription of genes involved in various group behaviors. SdiA is a LuxR-type receptor homolog found in *Salmonella*, *Escherichia, Klebsiella, Enterobacter*, and *Citrobacter* genera [14]. Interestingly, these species do not have LuxI-type synthases and do not produce AHLs; thus, SdiA represents an orphan [14] or “solo” LuxR-type receptor, a class that is rapidly growing in number [15]. SdiA from the common foodborne pathogen, *S. enterica* serovar Typhimurium (*S.* Typhimurium hereafter), has been a target of research [16–19] and has high sequence identity with SdiA from other genera: for example, *S.* Typhimurium (GeneBank AAC08299.1) SdiA is 72% identical to *Escherichia coli* (GeneBank AWF10864.1) SdiA, 67% identical to *Klebsiella pneumoniae* (GeneBank CDO1572.1) SdiA, 71% identical to *Enterobacter clocae* (GeneBank AFN80302.1) SdiA, and 84% identical to *Citrobacter koseri* (GeneBank SQB29462.1) SdiA.

Early studies of SdiA in *S.* Typhimurium and *E. coli* identified low levels of AHL-independent SdiA activity by overexpressing SdiA from a plasmid [23–26]. However, once Michael et al. [16] discovered that SdiA in *S.* Typhimurium responds to exogenously supplied natural AHLs, AHL-dependent SdiA regulons were identified in both *S.* Typhimurium [27–28] and *E. coli* [14,29–31]. In *S.* Typhimurium, SdiA promotes transcription of *pefI*, *srgA*-*E*, and *rck* [28,32]. *pefI* and *srgA* are members of the plasmid encoded fimbriae (pef) operon; PefI negatively regulates the pef operon and SrgA is a disulfide oxidoreductase involved in correctly folding PefA, a fimbrial subunit [33]. The functions of SrgB-D are unknown [28]. SrgE is a type III secreted effector, but its target is unknown [19]. Lastly, rck (resistance to complement killing) has two known functions that are critical for infection: rck confers resistance to human complement and is responsible for the zipper mechanisms by which *S.* Typhimurium invades host cells [34–35].

AHLs produced by other species in the *Salmonella* and *E. coli* environment are believed to be critical to SdiA function. For example, SdiA in *S.* Typhimurium has been shown to be activated in the presence of AHL-producing pathogens in the digestive tract of mice (producing organism: *Yersinia enterocolitica*) [18] and turtles (producing organism: *Aeromonas hydrophila*) [17]. Further, the introduction of a LuxI-type synthase (via a plasmid) into *S.* Typhimurium provided the pathogen with a competitive advantage in colonizing mice over bacteria that lacked the plasmid [18]. Similarly, enterohemorrhagic *E. coli* (EHEC) requires SdiA and AHLs produced by other species in the bovine rumen in order to colonize cattle [19,36]. These results strongly indicate the importance of SdiA in the virulence of this family of pathogens. Despite these findings, however, the precise roles of SdiA in QS and in promoting survival and host colonization remain poorly understood.

What we lack in mechanistic understanding of SdiA’s role in virulence is perhaps made up for by the ability to produce and manipulate SdiA in vitro. Indeed, SdiA appears to be far more stable and amenable to characterization in vitro relative to other LuxR-type receptors, and is poised for biophysical characterization [21–22,37]. LuxR-type proteins consist of two domains: a larger N-terminal ligand-binding domain (LBD) connected to a smaller C-terminal DNA-binding domain (DBD). In 2006, the structure of the EHEC SdiA LBD was solved by NMR in the presence and absence of AHL and demonstrated increased folding and structure upon ligand binding [37]. Recently, two groups reported X-ray crystal structures of full-length EHEC SdiA as a homodimer in the presence of four naturally occurring AHLs (shown in Figure 1B) [21–22]. These studies reveal a structure for the SdiA dimer that incorporates LBD and DBD domains comparable to those of the other reported full length LuxR-type proteins (i.e., TraR and QscR) [21–22], albeit with different interdomain interactions that likely direct the final assembly. Despite these reported structures, we still have a very poor understanding of non-native ligand–receptor interactions involved in LuxR-type receptor activation (or inactivation). Most LuxR-type proteins are highly unstable in vitro in the absence of an agonist ligand, and this instability is typically heightened in the presence of an antagonist [38]. As such, the observed stability of EHEC SdiA in vitro, both in the absence and presence of AHLs, provides a new and potentially powerful pathway to begin to delineate the AHL:LuxR-type receptor interactions that engender agonism, and possibly, antagonism [21–22]. Such studies will require AHL-type ligands capable of SdiA agonism and antagonism.

Non-native ligands that modulate the activity of many different LuxR-type receptors have been utilized to delineate the mechanism of various QS systems, to understand the roles of QS in infection, and to attenuate virulence phenotypes in wild-type bacteria in the absence and presence of their native hosts [38–50]. The majority of these compounds have been based on the AHL scaffold. The development of small molecule probes for SdiA has lagged relative to these prior studies. Indeed, to our knowledge, there have only been two reports of experimental studies of AHL-type ligand activity in SdiA in any bacterial species, and no antagonists have been reported. The first study involved the discovery by Michael et al. [16] that *S*. Typhimurium actually responds to exogenous natural AHLs (vide supra), demonstrated through the testing of the C4, C6, C8, C10, and C12 AHLs as well as their 3-oxo analogs. In 2007, Janssens et al. characterized the potency of a small set of AHLs (as racemic mixtures) in SdiA from *S.* Typhimurium with varied tail lengths (4–14 carbons), oxidation levels at the tail β-carbon, and lactone head group replacements [20]. One AHL, *N*-(3-oxooctanoyl) DL-homoserine lactone (DL-OOHL, shown in Figure 1B) was determined as the optimal natural AHL for SdiA (EC_50_ = 3 nM, as determined using a cell-based reporter for SdiA), and its homocysteine thiolactone analog (Figure 1B) was found to be three-fold more potent (EC_50_ = 1 nM). We sought to build on these prior studies in the current work and identify an expanded range of synthetic ligands for SdiA.

Herein, we report the screening of a focused library of AHL analogs for activity in the SdiA receptor from *S.* Typhimurium. Compound efficacies and potencies were measured in agonism and antagonism assays using an SdiA luminescence reporter system, and follow-up studies were performed in an *E. coli* SdiA reporter. The results provide a broad picture of the types of AHL scaffolds that can agonize and antagonize *S.* Typhimurium SdiA, allowing for the definition of key structure–activity relationships (SARs) for the modulation of SdiA activity. These compounds represent new chemical tools for exploring the role of SdiA and QS in *S.* Typhimurium infections, for characterizing the mechanisms by which non-native AHLs interact with LuxR-type proteins, and for developing pathways toward novel antivirulence strategies targeting SdiA.

## Results and Discussion

**Selection of the AHL library for screening.** We sought to examine a range of AHL-type scaffolds for activity in SdiA. We selected a series of sub-libraries from our in-house compound collections for analysis with demonstrated activities in other LuxR-type receptors, including TraR from *Agrobacterium tumefaciens* [45,51–54]; AbaR from *Acinetobacter baumannii* [47,55]; LasR [45,51–54,56], QscR [57], and RhlR [48,56] from *Pseudomonas aeruginosa*; ExpR1 and ExpR2 from *Pectobacterium carotovora* [46,58]; and LuxR from *Vibrio fischeri* [45,51–54]. The full set of 151 compounds tested is shown in Supporting Information File 1. An overview of the structures in each sub-library is provided below.

Sub-libraries A and H contained AHLs with differing acyl tail lengths and oxidation levels at the tail β-carbon, including many naturally occurring AHLs [51,55]. The B and D sub-libraries were designed to test the effects of lactone stereochemistry, substitution of a variety of more structurally diverse and non-native functional groups on the acyl tail (e.g., alkyl, cycloalkyl, and aryl), and alkyl linker length between the head group and these functional groups [51]. The C and E sub-libraries consisted of substituted phenylacetanoyl homoserine lactones (PHLs), phenylpropionyl homoserine lactones (PPHLs), and phenoxyacetyl homoserine lactones (POHLs), many of which we have previously found to be highly active in a range of LuxR-type receptors as both agonists and competitive antagonists [45,47–48,51,56]. The Q and R sub-libraries contain a related group of substituted benzoyl homoserine lactones (BHLs) [56]. Sub-library S probed the effects of branched alkyl groups on the acyl tail [56]. The F sub-library contained a variety of AHL analogs with alternative, often hydrolysis resistant head groups coupled to native-like alkyl tails, or aryl tails from known active PHLs or POHLs [53–54]. Notably, this sub-library contained a range of thiolactone analogs, including the L-OOHL thiolactone analog, for comparison to the work of Janssens et al. [20]. We also included a set of AHLs and non-AHL-derived compounds (termed “library **1–22**”) that have been reported by our laboratory and others to be strong modulators of LasR in *P. aeruginosa* [59]. As these compounds represent some of the best-characterized LuxR-type receptor modulators reported, the examination of their activity profiles in SdiA was also of interest. Lastly, we included compound **23**, 1-octanoyl-*rac*-glycerol (Figure 1B), in our assays as X-ray crystallographic studies revealed it was present in the AHL binding site of SdiA (from EHEC) when purified in the absence of AHL (i.e., a complex that originally was thought to be “apo” SdiA [22]), and we sought to determine if it had any functional activity in SdiA.

**Biological assays for SdiA activity.** Cell-based reporter strains that rely on detecting the transcriptional activity of LuxR-type receptors are commonly used to assess the activity of exogenous ligands. We used the *S.* Typhimurium-pJNS25 reporter strain constructed by Smith and Ahmer [27] and also used by Janssens et al*.* [20] to examine SdiA activity. This strain naturally produces SdiA and contains a reporter plasmid with the promoter region for *srgE* fused to the *luxABCDE* operon of *V. fischeri* (see Experimental section for details of all strains and plasmids). SdiA activity is thus reported by the production of luciferase and resulting bioluminescence. We also prepared an SdiA reporter in *E. coli* (JLD271-pJN105SE-pSC11SE) to examine *S.* Typhimurium SdiA activity in an alternate background and with a different reporter gene output. *E. coli* JLD271 is the *sdiA* mutant of K-12 *E. coli* [60]. This reporter construct uses an arabinose inducible promoter to produce *S.* Typhimurium SdiA and the promoter region for *srgE* fused to *lacZ* to report SdiA activity. SdiA activity is then measured using standard β-galactosidase assays [61]. In both reporter strains, signal was normalized to the difference between the positive control (10 μM OOHL) and the negative control (1% DMSO; no compound).

We note that, due assumedly to its enhanced stability relative to other LuxR-type receptors, *S.* Typhimurium SdiA was observed to have activity in these reporter assays even in the absence of exogenous AHL, leading to a higher background signal from the negative control relative to that typically observed in LuxR-type receptor reporter assays [38,59]. For the *S.* Typhimurium reporter, the negative control was at 20% the level of the positive control based on raw luminescence. Further, for the *E. coli* JLD271 reporter, the negative control was at 50% the level of the positive control; we reason that this higher background relative to *S.* Typhimurium is due to the overexpression of SdiA in this reporter. Conditions for both assays (length of incubation, temperature of incubation, and β-galactosidase substrate for developing the *E. coli* assay) were carefully optimized to maximize the signal-to-noise ratio between the positive and negative control in view of these high background levels (data not shown) [62].

**Figure 2:**
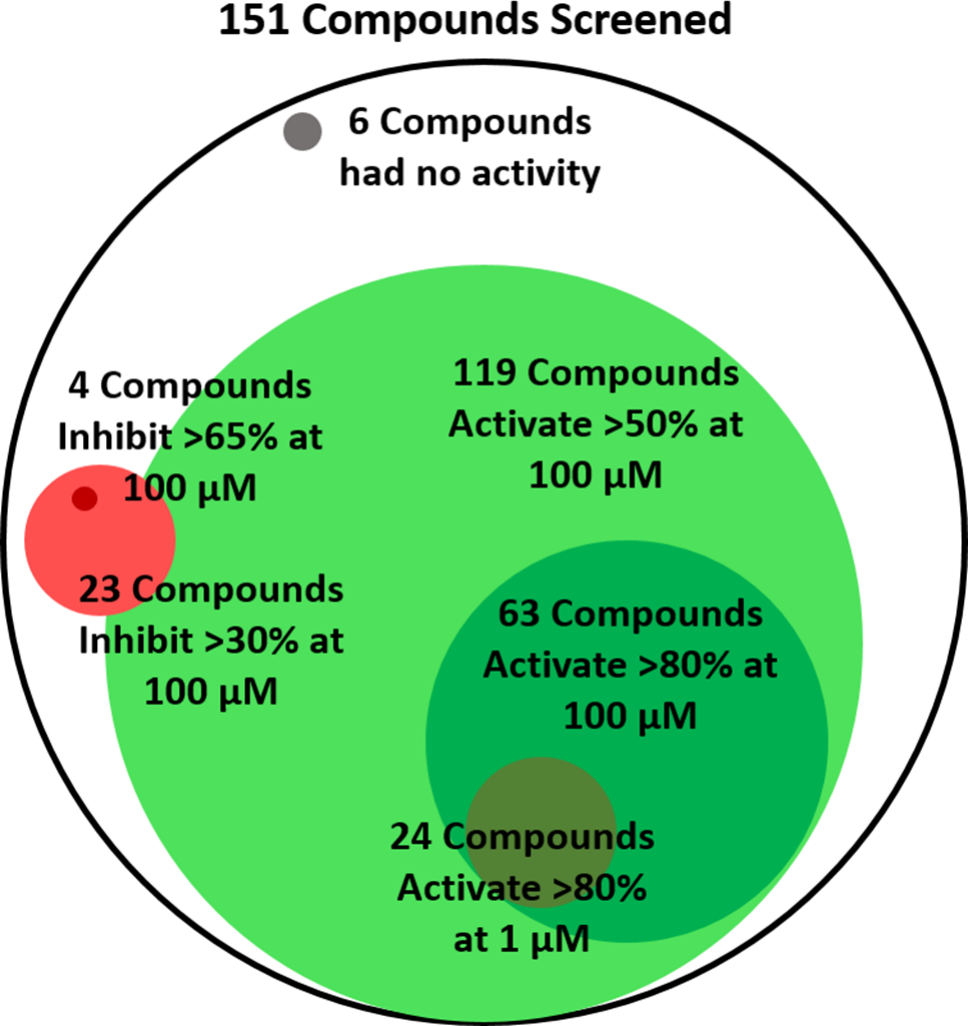
Overview of SdiA agonism and antagonism single-point screening results in the *S.* Typhimurium reporter. Agonists are indicated in green. Antagonists are indicated in red. Compounds with less than 10% agonism and antagonism (i.e., no activity) are indicated in grey. All circles are scaled to their proportion of the library. Overlaps largely indicative of overlapping compound sets.

**Initial screening results in the *S.* Typhimurium SdiA reporter.** All 151 compounds were screened for agonism (at 100 μM and 1 μM) and competitive antagonism (at 100 μM and 1 μM) in the *S.* Typhimurium SdiA reporter. The full assay results are tabulated in Supporting Information File 1, and an overview is provided in Figure 2. For agonism at 100 μM, 119 compounds (79% of the library), and at 1 μM, 71 compounds (47% of the library), activated SdiA by at least 50% (above the negative control). This level of promiscuity in terms of agonist ligands is high for a LuxR-type receptor. For comparison, RhlR and QscR were activated beyond 50% by only 23% [56] and 11% [57] of a comparable in-house library at 100 μM and 5 μM, respectively. General trends for SdiA agonism are listed here. All natural AHLs with acyl tail lengths of 4–12 carbons, regardless of the oxidation state at the β-carbon, were able to activate SdiA by greater than 50% at 100 μM. Most of the PHLs, PPHLs, and POHLs were able to activate SdiA more than 50% at 100 μM, but BHLs were not as well tolerated. The D sub-library results suggested that a wide range of functional groups and multiple ring systems could be tolerated on the acyl tail. Changing the head group to a phenyl or cyclohexane was not well tolerated; however, cyclopentane, thiolactones, and the alternative stereochemistry, D-lactone were generally tolerated. Interestingly, 1-octanoyl-*rac*-glycerol (**23**) showed no agonism in this reporter assay, suggesting it does not act as an AHL signal surrogate in SdiA [22]. At 1 μM, only 24 compounds (16% of the library) were able to activate SdiA greater than 80%. To narrow this study, these 24 compounds were selected for further characterization to determine their relative potencies.

For the SdiA antagonism assays, compounds (at 100 μM) were competed against the EC_90_ of (enantiopure) L-OOHL (10 nM). Only 4 compounds (3% of the library) were able to inhibit SdiA activity in *S*. Typhimurium by greater than 65% under these conditions (Figure 2). This is a lower percentage of inhibitors than we typically identify for this AHL analog library, even with the heightened stringency of testing against an agonist (here, L-OOHL) at its EC_90_ value. For reference, 24% and 12% of a comparable in-house library were found to inhibit QscR [57] (at 5 μM) and RhlR [56] (100 μM) by greater than 65%, respectively. Lowering our cut-off, we found 23 compounds that could inhibit SdiA by greater than 30%. These compounds could be classified as follows: long chain AHLs (12–16 carbons); BHLs, PHLs and PPHLs with large substituents on the aryl ring; glycine ethyl ester replacements for the lactone head group; and compound **11** (ITC-12), originally reported by the Meijler lab [44], which has an isothiocyanate (itc) at its acyl tail terminus installed for potential covalent capture in the AHL-binding site (vide infra). These 23 compounds were selected for further characterization to determine their relative inhibitory potencies in SdiA.

**Figure 3:**
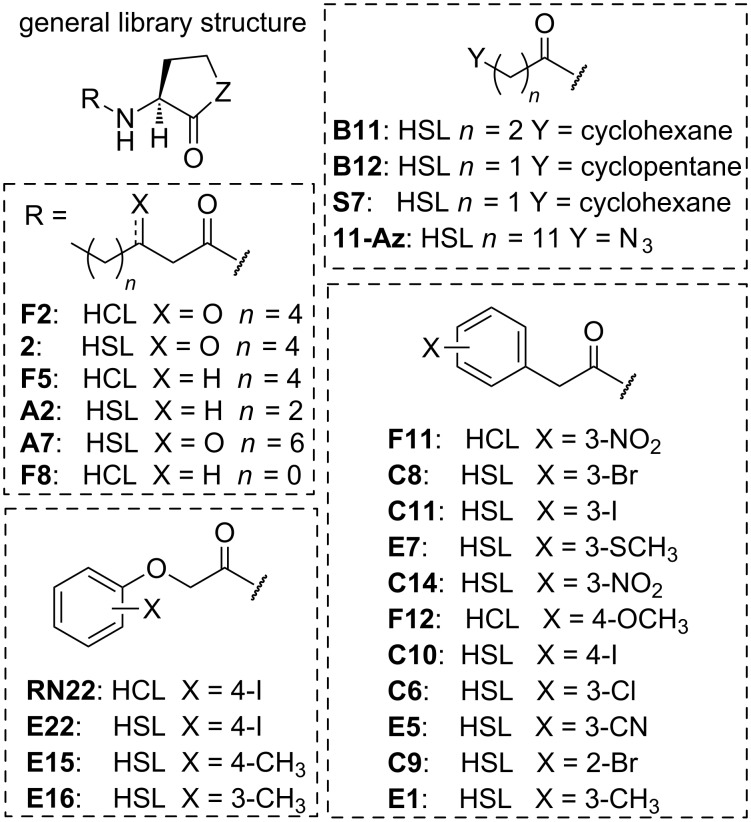
Chemical structures of the most potent SdiA agonists. Compound names (except for **11-Az**) match those reported in our prior publications. HSL = homoserine lactone, Z = O. HCL = homocysteine thiolactone, Z = S. Compound **2** = L-OOHL. Compounds within each cluster (indicated by hashed line box) are listed in order of highest to lowest potency.

**Characterization of the efficacies and potencies of SdiA agonists.** The lead agonists were subjected to dose–response analysis using the *S.* Typhimurium SdiA reporter as described in the Experimental section (see Supporting Information File 2 for the full dose–response curves). The structures of the agonist compounds are shown in Figure 3 and their maximal activities (i.e., efficacies) and EC_50_ values (i.e., potencies) are listed in Table 1.

**Table 1:** Characterization data for SdiA agonists (listed by potency).^a^

compound	activation (%)	EC_50_ (nM)	95% CI (nM)^b^

**F2**	119	0.70	0.39–1.25
**2** (L-OOHL)	110	1.03	0.549–1.92
**F5**	79^c^	2.28	1.42–3.67
**F11**	97	2.47	1.49–4.08
**RN22**	93	6.08	3.89–9.49
**E22**	108	10.4	6.68–16.1
**C8**	96	19.1	13.8–26.3
**C11**	90	20.6	15.8–26.8
**A2**	81^c^	20.9	18.0–24.3
**E7**	91	26.0	17.8–38.0
**A7**^d^	85^c^	26.6	22.3–31.7
**E15**	98	33.0	20.6–52.9
**F12**	84	38.8	21.4–70.4
**E16**	100	43.3	33.1–56.8
**C10**	67^c^	47.2	35.7–62.3
**B11**	93	50.1	36.7–68.5
**C6**	95	51.9	38.8–69.6
**B12**	93	54.7	43.9–68.2
**E5**	73^c^	62.2	50.6–76.6
**F8**^d^	91	62.8	56.4–70.0
**C9**	103	63.5	50.4–80.1
**E1**	99	66.4	48.3–91.2
**S7**	89	106	60.7–185
**11-Az**	110	125	82.5–190

^a^All assays are biological triplicates of technical triplicates using the *S.* Thyphmurium-pJNS25 reporter strain (see Experimental section). ^b^CI = confidence interval for the EC_50_ value. ^c^Maximal activity was significantly (*p* = 0.05) different than the positive control, 10 μM L-OOHL (**2**). See ref. [62]. ^d^Hill slope in dose response curve significantly different from 1 (*p* = 0.05). **A7** = 0.84 +/− 0.05 (SD); **F8** = 1.24 +/− 0.07 (SD).

Corroborating prior work by Michael et al. [16] and Janssens et al. [20], SdiA was found to be most strongly activated by natural AHLs with a six to eight carbon tail (**2**, **A2**; Figure 3). The PHL class was also highly active in SdiA; of the 36 PHLs tested, 30 showed greater than 75% activity at 100 μM. Based on potency, PHLs with a *meta* substitution were favored for SdiA agonism (**F11**, **C8**, **C11**, **E7**, **C14**, **C6**, **E5**, and **E1**), but certain *para* (**C10**, **F12**) and *ortho* (**C9**) substituted PHLs were also highly potent. The nature of the substituent at the *meta* position could vary, ranging from electron withdrawing (NO_2_, **F11**) to electron donating (SCH_3_, **E7**), but larger substituents were favored (e.g., I and Br over Cl). Many of these highly potent PHL agonists of SdiA are also potent antagonists of other LuxR-type receptors, most notably *para*-iodo-PHL **C10**, which inhibits RhlR [56], AbaR [47], LuxR [45], ExpR1 [58], ExpR2 [58], and TraR [45]. Strikingly, the top agonist PHL structures identified for SdiA are similar to those in RhlR; six of the eight PHL SdiA agonists characterized are also RhlR agonists [56]. This correlation is interesting because *sdiA* is the descendent of a horizontal gene transfer of *rhlR* [63]. Indeed, the sequence of SdiA from *Salmonella* is 45% identical to RhlR, more than it is to LasR (27%), QscR (33%), TraR (23%), LuxR (27%), or CviR (32%) [21]. In further support of a possible similarity between SdiA and RhlR, the cognate AHL signal of RhlR, butanoyl HL **A1**, is also a moderate agonist of SdiA, with 100% maximal activation and an EC_50_ of 578 nM (see Supporting Information File 2 for full dose–response curve).

The most intriguing class of SdiA agonists was the POHLs. All three of the POHLs tested (**E22**, **E15**, and **E16**) tested and one thiolactone POHL analog (**RN22**) activated SdiA by more than 75% at 1 μM. This finding is in stark contrast to our previous studies of POHLs in other LuxR-type receptors, as we have largely only found them to be antagonists. For instance, *para-*iodo **E22**, the most potent POHL agonist in SdiA, is an antagonist in RhlR [56], TraR, LuxR, and LasR [45]. POHL structures were not as well sampled in the assembled compound library as PHLs; these data suggest the screening of additional POHLs in SdiA in the future to better delineate SARs for this structure class. Overall, the agonism reporter assay data support SdiA as a less selective receptor for AHL-type agonists (assuming these non-native ligands target the AHL-binding site). Perhaps more interestingly, as SdiA appears to be activated strongly by non-native AHLs that typically inhibit other LuxR-type receptors, these data underscore the potential value of SdiA as a useful model system for investigating the interactions these ligands can have with LuxR-type receptors in vitro.

Only one class of alternate head group-containing compounds was present in the set of top SdiA agonists, the homocysteine thiolactone, corroborating prior work by Janssens et al. [20]. Because several thiolactone compounds (**F2**, **F5**, **F11**, **RN22**, **F12** and **F8**; structures shown in Figure 3) were quite potent (EC_50_ values ranging from 0.7–91 nM), we sought to characterize their lactone analogs (structures shown in Supporting Information File 1) to determine if the thiolactone substitution results in increased potency (comparison data in Table 2, see Supporting Information File 2 for full dose–response curves). While the potencies of thiolactones **F2** and **RN22** were not significantly different than their lactone analogs **2** and **E22**, the simple alkyl derivatives (thiolactones **F8** and **F5** vs lactones **A1** and **A3**) and the PHL-type derivatives (thiolactones **F11** and **F12** vs lactones **C14** and **C24**) were nearly an order of magnitude more potent. As thiolactone hydrolysis can occur more slowly than lactone hydrolysis in the AHL scaffold [53], these compounds may find utility as chemical probes due to their longer half-lives in biological media. These results suggest that this structural motif could be installed to potentially improve the potencies of other lactone-based SdiA agonists and antagonists.

**Table 2:** Comparison of selected lactone and thiolactone analog EC_50_ values in SdiA.^a^

lactone	EC_50_ (nM)	95% CI (nM)^b^	corresponding thiolactone	EC_50_ (nM)	95% CI (nM)^b^	fold increase

**2** (OOHL)	1.03	0.55–1.92	**F2**	0.700	0.39–1.25	1.47^c^
**A1**	578	482–693	**F8**	62.8	56.4–70.0	9.20
**A3**	18.1	7.44–43.8	**F5**	2.23	1.42–3.67	8.12
**C14**	28.3	24.0–33.3	**F11**	2.47	1.49–4.08	11.5
**C24**	252	194–327	**F12**	38.8	21.4–70.4	6.49
**E22**	10.4	6.68–16.1	**RN22**	6.08	3.89–9.49	1.71^c^

^a^All assays are biological triplicates of technical triplicates using the *S.* Thyphimurium-pJNS25 reporter strain (see Experimental section). ^b^CI = confidence interval for the EC_50_ value. ^c^Not significantly different (*p* = 0.05).

**Figure 4:**
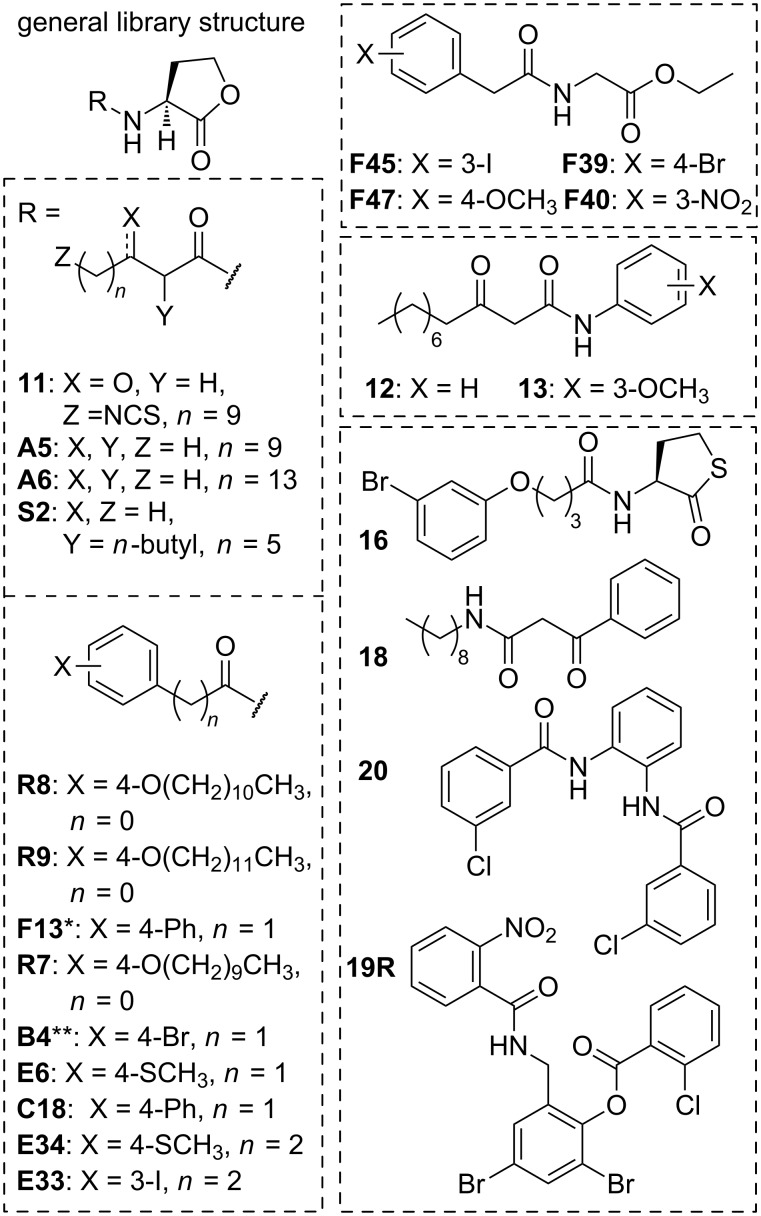
Chemical structures of the most potent SdiA antagonists. Compound names preceded by letters match those reported in our prior publications. * Indicates an L-thiolactone head group. ** Indicates a D-lactone head group. Compounds within each cluster (indicated by hashed line box) are listed in order of highest to lowest efficacy (maximal inhibition).

**Characterization of the efficacies and potencies of SdiA antagonists.** The lead antagonists identified in the single point screens were subjected to dose–response analysis using the *S.* Typhimurium SdiA reporter as described in the Experimental section (see Supporting Information File 2 for the full dose–response curves). The structures of the lead SdiA antagonists are shown in Figure 4 and their maximal inhibition and IC_50_ values are listed in Table 3.

**Table 3:** Characterization data for SdiA antagonists (listed by efficacy).^a^

compound	inhibition (%)	activation (%)	IC_50_ (μM)	95% CI (μM)^b^

**11**^c^	130^d^	−33	0.32	0.12–0.85
**R8**	120^d,e^	24	44.2	17.2–114
**16**^c^	89	48	44.6	11.7–17.0
**F45**	87	26	28.5	17.4–46.8
**R9**	63^e^	6	*–*^f^	*–*
**A5**	59^e^	57	*–*	*–*
**F39**	55^e^	50	*–*	*–*
**20**	53^e^	47	*–*	*–*
**A6**	52	20^e^	1.75	1.08–2.83
**F13**	51	59	2.35	0.781–7.08
**R7**	51	6^e^	3.10	1.57–4.18
**B4**	50^e^	52	*–*	*–*
**E6**	49^e^	74	*–*	*–*
**19R**^c^	44^e^	40	*–*	*–*
**F47**	42	61	*–*	*–*
**C18**	40^e^	48	*–*	*–*
**12**	39^e^	12	*–*	*–*
**18**	37^e^	53	*–*	*–*
**F40**	33	56	27.8	10.0–77.3
**S2**	32^e^	63	*–*	*–*
**E34**	30^e^	66	*–*	*–*
**E33**	19	67	9.64	3.25–28.6
**13**	19^e^	57	*–*	*–*

^a^All assays are biological triplicates of technical triplicates using the *S.* Thyphimurium-pJNS25 reporter strain (see Experimental section). ^b^CI = confidence interval for the IC_50_ value. ^c^Hill slope in dose response curve significantly different from −1 (*p* = 0.05). **11** = 0.40 +/− 0.08 (SD); **16** = −0.6 +/− 0.1 (SD); **19R** = −0.2 +/− 0.3 (SD). ^d^Inhibition is listed as 100% minus the lowest % activity observed in the presence of 10 nM L-OOHL (**2**). Values >100% inhibition suggest that AHL-independent (background) SdiA activity is being inhibited. ^e^Compound insolubility limited testing at higher concentrations; maximal observable activity reported. ^f^– = Non-linear fit to the data could not be completed.

Several structural classes of SdiA antagonists emerged from this study (Figure 4): long alkyl tail AHLs (**A5**, and **A6**) long alkyl tail-functionalized BHLs (**R7**, **R8**, and **R9**) PHLs and PPHLs with large aryl substituents (**B4**, **C18**, **E6**, **E33**, **E34**, and **F13**), and PHL-type derivatives with glycine ethyl ester head groups (**F39**, **F40**, **F45**, and **F47**). In addition, an AHL with an electrophilic warhead for covalent modification (**11**, ITC-12) and various non-AHL compounds (**12**, **13**, **18**, **19R**, and **20**) were found to be SdiA antagonists. Most of the compounds that displayed antagonistic behavior were actually classical partial agonists of SdiA (i.e., capable of activating SdiA to a lower maximal level, and then inhibiting to that same level when competed against OOHL (**2**) [59]); maximal inhibition and maximal activation are both listed in Table 3 to underscore this activity profile. We note that **11** and **R8** were observed to inhibit SdiA beyond 100% in these competitive antagonism assays. We speculate that this activity profile is due to the ability of these compounds to inhibit both AHL-dependent and AHL-independent SdiA activity, and return to this below.

Both C12 AHL **A5** and C16 AHL **A6** inhibited SdiA activity by greater than 50%. Interestingly, C14 AHL **H26** agonized SdiA to 39% (at 100 μM), but failed to inhibit SdiA (see Supporting Information File 1). Further, the 3-oxo analogues of **A5** and **A6** displayed SdiA agonism: **1** (3-oxo-C12) fully activated SdiA at 100 μM and **H25** (3-oxo-C16) activated SdiA to 57% at 100 μM, suggestive that contacts with the 3-keto group are important for receptor agonism. Antagonists **R7**, **R8**, and **R9** are all BHLs with long alkyl tails (10, 11, and 12 carbons, respectively) in the *para* position. Of these three compounds, **R8** displayed the greatest inhibitory activity in SdiA – inhibition to 120% with an IC_50_ of 44 μM. Interestingly, similar compounds with shorter tail lengths are potent inhibitors in other receptors: **R6**, with a 9-carbon tail, is a potent inhibitor of QscR and LasR, and **Q9**, with an 8-carbon tail, is a potent inhibitor of QscR [64]. A set of other compounds containing aryl tails with large substituents (such as Br, I, and SCH_3_) also partially inhibit SdiA; specifically, thiolactone **16** (mBTL), which has a long (4 atom) linker between the amide and phenyl, inhibited SdiA activity by 89% with an IC_50_ of 45 μM. This compound was reported by the Bassler lab to also display partial agonism in RhlR in *P. aeruginosa* [50].

The defining features of these first three classes of SdiA antagonists are their relatively large tail groups. In the crystal structure of SdiA from EHEC bound to OOHL (**2**) [22], Nguyen et al. observed that two residues in the ligand-binding pocket, Phe59 and Leu77, were moved inward toward the alkyl tail of **2**, effectively closing the binding pocket relative to the “apo”-SdiA structure (bound to 1-octanoyl-*rac*-glycerol (**23**)). In *Salmonella* SdiA, these side chains are switched (Leu59 and Phe77), yet maintain bulky and hydrophobic character at these positions. We speculate that closing of this binding pocket on the AHL tail could differentiate the AHL-bound and highly active SdiA structure from the “apo” and less active SdiA structure. The sterically larger tails of the SdiA antagonists uncovered here could prevent the closing of the SdiA binding pocket and the transition to the more active state. While additional studies are clearly necessary to support this hypothesis, it is congruent with the SdiA structural data, the observation that 1-octanoyl-*rac*-glycerol (**23**) has no activity in the SdiA reporter assay, and recently reported data for LasR suggestive of a closed ligand-binding site for maximal activation [65].

All of the glycine ethyl ester head group compounds tested exhibited SdiA antagonism despite varying between a range of PHL- and PPHL-type tails with differing aryl substituents (Figure 4; Table 3). Prior studies of these compounds in our laboratory had shown they have minimal to low activity in LuxR-type receptors [54], so this activity profile in SdiA was unexpected. The *meta*-iodo PPHL derivative **F45** displayed the strongest antagonism of this structure class: 87% inhibition with an IC_50_ of 28.5 μM. Finally, itc-derivative **11** had the highest efficacy and potency of any SdiA antagonist reported herein. Compound **11** inhibited all AHL-dependent SdiA activity (and all of the AHL-independent SdiA (or background) activity; 130% effective inhibition) with a sub-micromolar IC_50_ (318 nM). Compound **11** was originally designed by the Meijler lab to react with a cysteine in the AHL-binding pocket of LasR, thereby acting as an irreversible inhibitor [44]. SdiA does have a cysteine in the binding pocket (Cys45, see Figure 1D), but it is positioned near carbons 3 and 4 of the acyl tail in OOHL (**2**) in the SdiA crystal structure, rather than near the terminus of the alkyl tail [22]. We examine the possibility of **11** covalently modifying SdiA as part of the additional biological assays described next.

**Heterologous SdiA reporter system and competition assay data.** We submitted the most efficacious antagonists from above (**11**, **16**, **R8**, and **F45**) to an *E. coli* SdiA reporter (JLD271-pJN105SE-pSC11SE) to further characterize their active profiles (see Experimental section for full assay details). Specifically, we sought to determine whether their apparent activities were due to directly inhibiting SdiA activity, indirectly inhibiting the *Salmonella* reporter by altering the level of SdiA produced, inhibiting the activity of the enzymatic reporter, luciferase, or some combination of these pathways. The assay results in the *E. coli* SdiA reporter are summarized in Table 4 and full dose–response curves are in Supporting Information File 2. Compounds **F45** and **16** failed to display antagonistic activity in the *E. coli* strain, while **11** and **R8** were able to fully inhibit SdiA activity (with **11** inhibiting most of the AHL-independent activity as well). Compound **11** retained its high potency in the *E. coli* reporter (IC_50_ = 380 nM); the IC_50_ for **R8** could not be determined due to solubility limitations at high concentrations. These results suggest that **R8** and **11** inhibit both the *E. coli* and *S*. Typhimurium reporters at the level of SdiA transcriptional activity, not by changing the expression of SdiA or by inhibiting the luminescence reporter. Conversely, the inability of **F45** and **16** to even partially inhibit SdiA in the *E. coli* reporter system indicates that the means by which they inhibit SdiA activity in the *S.* Typhimurium reporter is dependent on either the expression of SdiA, the luminescence reporter system, or other *Salmonella* specific targets that could alter SdiA activity. None of these possible mechanisms of action would be ideal for probing AHL-mediated SdiA activity, and these follow-up studies underscore the value of using a heterologous strain to validate the activity of possible LuxR-type receptors modulators.

**Table 4:** SdiA antagonism assay data for select compounds in the *E. coli* reporter.^a^

compound	inhibition (%)	IC_50_ (nM)	95% CI (nM)^b^

**11**	130^c^	380	175–822
**R8**	106	–^d^	–
**16**	NA^e^		
**F45**	NA		

^a^All assays are biological triplicates of technical triplicates using the *E. coli* JLD271-pJN105SE-pSC11SE reporter system (see Experimental section). ^b^CI = confidence interval for the IC_50_ value. ^c^Inhibition greater than 100% suggests inhibition of AHL-independent SdiA activity. ^d^– = Non-linear fit to the data could not be completed. ^e^NA = not active.

**Figure 5:**
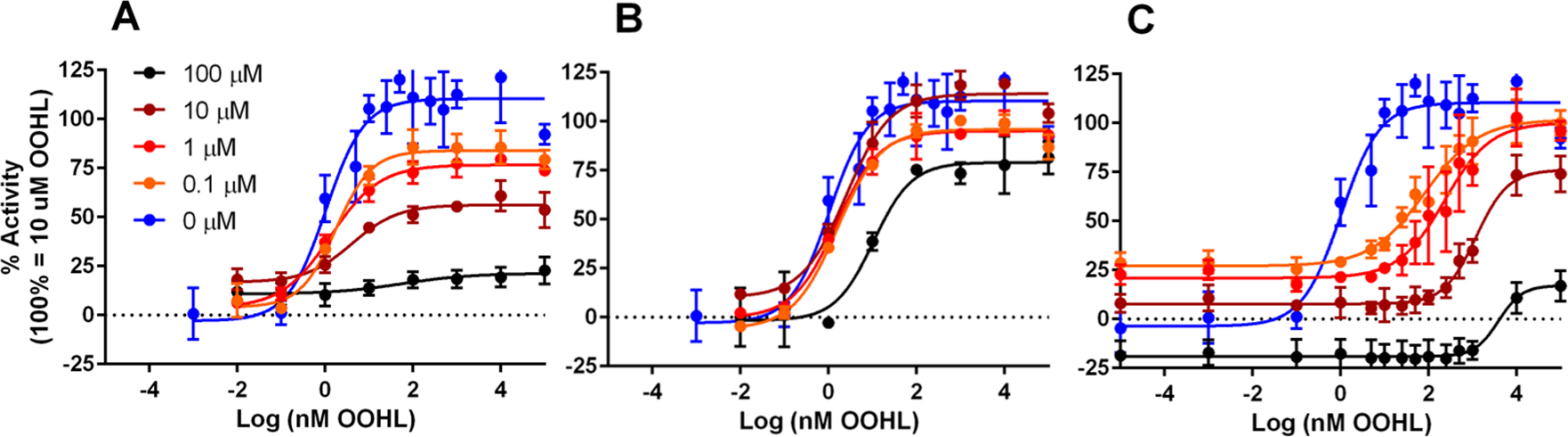
Dose–response activity curves for OOHL (**2**) in competition against various concentrations of synthetic compounds in the *S.* Typhimurium SdiA reporter. (A) Data for compound **F45**, (B), **R8**, and (C) **11**. Figure legend in panel A applies to panels B and C. All assays are biological triplicates of technical triplicates. See Experimental section for full assay details.

We also submitted compounds **11**, **R8**, and **F45** to competition-type assays in the *S.* Typhimurium SdiA reporter to further characterize their activity profiles. (Because thiolactone **16** displayed multimodal, or non-monotonic [59] activity in the *S.* Typhimurium SdiA reporter (agonism at low concentrations and antagonism at high concentrations; see Supporting Information File 2) and did not display any activity in the *E. coli* reporter, we chose not to include it in this initial competition analysis.) In the competition assay, varied concentrations of the antagonist were competed against OOHL (**2**) in a dose–response-type analysis. If the EC_50_ of OOHL increased with increasing concentration of antagonist, yet its maximal activity was maintained, these results would be supportive of the compound acting as a competitive antagonist of SdiA in the reporter assay. However, if the maximal activity of OOHL decreased with the concentration of added antagonist, these results would be supportive of the compound acting as a non-competitive antagonist of SdiA. The results are shown in Figure 5 (EC_50_ and maximal activity values listed in Supporting Information File 3). The glycine ethyl ester **F45** showed a non-competitive inhibition profile in these competition assays (decreasing the maximal activity to 20% in the presence of 100 μM **F45**, Figure 5A), and as highlighted above, failed to inhibit SdiA in the *E. coli* reporter. These results support a reporter dependent, rather than SdiA dependent, inhibitory activity for **F45**. BHL **R8**, which was an SdiA antagonist in both reporter systems, yielded dose–response curves against OOHL (**2**) largely supportive of competitive SdiA inhibition in the *S.* Typhimurium reporter, increasing the EC_50_ of OOHL from 1.03 nM to 10.6 nM with only a small decrease in maximal activity to 80% (Figure 5B). These results support the conclusion that **R8** inhibits SdiA activity by targeting SdiA directly.

Itc-derivative **11** showed both competitive and non-competitive inhibition of OOHL (**2**) in the competition assay (Figure 5C): the EC_50_ increased from 1.03 nM to 4.67 μM while the maximal activity decreased to 20%. This activity trend is consistent with **11** interacting with SdiA both reversibly (by presumably outcompeting OOHL in the ligand binding site) and irreversibly (by covalently modifying SdiA). Such a dual-activity mechanism was previously reported by the Meijler lab for **11** in LasR [44]. Because AHL-dependent and AHL-independent inhibition was observed in both reporter systems, it is likely that **11** targets SdiA for covalent modification rather than some other target that affects the reporter system. To further probe the hypothesis that **11** binds competitively to the SdiA ligand-binding site, we examined the structurally homologous, yet unreactive azide analog of **11**, **Az-11** (structure shown in Figure 3), in the *S.* Typhimurium SdiA reporter. **Az-11** was found to fully agonize SdiA with an EC_50_ of 125 nM (Table 1), in contrast to the full antagonism and 318 nM IC_50_ of **11** in SdiA, providing indirect support that **11** could bind in the AHL-binding site. Further experiments are needed to characterize the mechanism of SdiA inhibition by **11**, and are on-going.

## Conclusion

In summary, a focused library of non-native AHL analogs was screened in a cell-based reporter strain for agonism and antagonism of the LuxR homolog, SdiA, from *S.* Typhimurium. This AHL library contained many scaffolds with demonstrated agonism and antagonism activity in other LuxR-type receptors. Despite the relative structural diversity of the library, nearly 80% of the compounds were able to activate SdiA by more than 50% at 100 μM. This level of promiscuity in terms of agonist ligand is extreme in comparison to other well-studied receptors, such as LasR. The most potent agonists of SdiA were found to be AHLs with medium length acyl tails (7–8 carbons), PHLs with *meta* substituents, and almost all of the POHLs tested. This study further underscores the “privileged” nature of the PHL structural class as potent ligands for LuxR-type receptors, and provides strong support for further analysis of the POHL class. In almost every case, the substitution of a thiolactone for the lactone head group increased the potency of the compound either as an agonist or as an antagonist of SdiA.

The SdiA antagonists uncovered herein all had relatively bulky acyl tails, suggestive that sufficient bulk on the AHL ligand can deactivate SdiA activity. In fact, the majority of these compounds were actually partial agonists of SdiA, but three types of antagonists had the ability to fully inhibit SdiA in the *S.* Typhimurium reporter system: BHLs with large alkyl substituents exemplified by **R8**, glycine ethyl ester head groups exemplified by **F45**, and the itc-functionalized compound **11**. Analyses of these antagonists in a heterologous SdiA reporter system and additional competition assays against OOHL (**2**) supported the conclusion that **R8** and **11** directly inhibit SdiA activity. **R8** acts by a competitive inhibition mechanism, while **11** displays a pattern of activity suggestive of both reversible and irreversible inhibition of SdiA.

There were several important outcomes of this study. First, this work provides the first set of chemical probes for SdiA, with a broad range of agonistic and antagonistic activities, which will provide a new entry into the study of QS in *S.* Typhimurium and its role in infections. Second, we have found that many of the ligands identified herein as active in SdiA are also potent agonists and antagonists of other LuxR-type receptors. In view of the heightened stability of SdiA in vitro relative to these other receptors, coupled with this overlap of active ligands, we believe that the biophysical bases of these agonistic and antagonistic activities can now be explored in vitro using SdiA, for the first time, to improve the fundamental understanding of the modes by which ligand binding modulates LuxR-type receptor activity. Third, several tactics have been identified that can be used for developing second-generation AHL-type ligands with enhanced potencies in SdiA: using electrophilic groups to target the cysteine in the SdiA binding pocket (taking a possible cue from **11**); delineating the SARs for activity by the POHL class, with an eye toward examining bulky substituents that should engender antagonism; and the incorporation of thiolactone head groups into lead compounds. Fourth and finally, in view of the close homology of the known SdiA receptors in *Escherichia, Klebsiella, Enterobacter*, and *Citrobacter* genera, the compounds and tactics reported herein should be exportable to these other bacteria, thereby significantly expanding their utility as chemical approaches to study QS.

## Experimental

**Bacterial strains and growth conditions.** The strains, plasmids, and primers used in this study are summarized in Table 5. All biological media and reagents were obtained from commercial sources and used according to the manufacturers’ instructions. All strains were grown in lysogeny broth (LB) at 37 °C with shaking (at 200 rpm). Bacterial growth was assessed by measuring cell culture density by absorbance at 600 nm (OD_600_). *S.* Typhimurium-pJNS25 was grown in 20 μg/mL tetracycline. *E. coli* JLD271-pJN105SE-pSC11SE was grown in 10 μg/mL gentamicin and 100 μg/mL ampicillin.

**Table 5:** Bacterial strains, plasmids, and primers used in this study.^a^

strain, plasmid, or primer	description	reference

*E. coli* DH5α	F^−^, Δ80d*lacZ* Δ M15 Δ (*lacZYA-argF*)U169 *deoR recA*1 *endA*1 *hsdR*17(rk^−^, mk^+^) *phoA supE*44 λ^− ^*thi*-1 *gyrA*96 *relA*1	Invitrogen
*E. coli* JLD271	K-12 Δ*lacX74 sdiA271*::Cam; Cm^R^	[60]
*S.* Typhimurium 14028	wild type *S. enterica* serovar Typhimurium	ATCC
pJNS25	*psrgE-luxCDABE* transcriptional fusion reporter plasmid; Tet^R^	[27]
pJN105SE	arabinose-inducible expression plasmid for *sdiA*; Gm^R^	this study
pSC11SE	*psrgE-lacZ* transcriptional fusion reporter plasmid; Ap^R^	this study
*psrgE* forward primer	CATgtcgacCTGGTTAATGACGCGTGATACAGTCG	this study
*psrgE* reverse primer	CATggatccGGGAGAGCTAATTAGCTCTTTCAGG	this study
*sdiA* forward primer	CATgaattcATGCAGGAAAATGATTTCTTCACC	this study
*sdiA* reverse primer	CATgaattcATGCAGGAAAATGATTTCTTCACC	this study

^a^Abbreviations: Cm^R^, chloramphenicol resistance; Gm^R^, gentamicin resistance; Ap^R^, ampicillin resistance; Tet^R^, tetracycline resistance.

**Construction of *E. coli* JLD271-pJN105SE-pSC11SE.** The same promoter region used by Smith and Ahmer [27] to construct pJNS25 was used for the promoter region in pSC11SE [66]. 477 base pairs of the *srgE* (STM1554) promoter region (−330 to +147) from *S.* Typhimurium (14028) were cloned into pSC11, using the *psrgE* primers listed in Table 5 (cut sites are lowercase). 723 base pairs of *sdiA* from *S.* Typhimurium were cloned into pJN105, using the *sdiA* primers listed in Table 5 (cut sites are lowercase) [66]. PCR-generated fragments were digested, ligated with cut vector, and transformed into *E. coli* DH5α using standard restriction digest cloning methods as we have reported previously [67]. These plasmids were then transformed into *E. coli* JLD271.

**Chemistry and compound handling.** The compounds tested were either synthesized as previously described [44–45,53–54,57,64] or purchased. Compounds **1**, **2**, **3** and **A1** were purchased from Sigma-Aldrich. The *E. coli* reporter assay substrate, *ortho*-nitrophenyl-β-D-galactopyranoside (ONPG), was purchased from DOT Scientific. Stock solutions of compounds were prepared at 10 mM in DMSO and stored at −20 °C in sealed vials.

***Salmonella* SdiA reporter assay. ***S.* Typhimurium-pJNS25 was grown 16–18 h overnight in LB, diluted 1:100 in fresh LB medium, and then incubated for 6 h at 37 °C with shaking at 200 rpm in the presence of compounds (1% DMSO). For antagonism assays, OOHL (**2**) at its EC_90_ (10 nM, 0.1% DMSO) was added to the subculture immediately prior to adding the compound for testing. All compounds were screened at 100 μM and 1 μM in triplicate in agonism and antagonism assays. Raw luminescence values were divided by the OD_600_ and then normalized to the controls; negative (DMSO) and positive (10 μM OOHL) control samples were included in every assay plate and used to normalize assay results, setting the positive control to 100% and the negative control to 0%. Luminescence was measured using a Biotek Synergy 2 plate reader and Gen 5 software (version 1.05). Dose–response analyses were performed by preparing dilutions of compounds in DMSO and testing each concentration in the agonism or antagonism assays. Competition dose–response assays (data in Figure 5) were performed in the same manner as the antagonism assays, except instead of OOHL being added to the subculture, the antagonist being tested was added. Non-linear regression curve fits were generated using GraphPad Prism software (version 6) using variable slope (four parameter) dose–response analysis.

***E. coli* SdiA reporter assay.** The β-galactosidase assay using the *E. coli* SdiA reporter was performed as reported previously, with minor modifications [56,61]. The reporter strain was grown 16–18 h overnight in LB, diluted 1:10 in fresh LB medium, and incubated at 37 °C with shaking at 200 rpm until it reached an OD_600_ of 0.25. Expression of SdiA was induced by the addition of 4 mg/mL arabinose, and the culture was incubated in the presence of compounds (1% DMSO) for 4 h at 37 °C with shaking at 200 rpm. For antagonism assays, the subculture was supplemented with OOHL (**2**) at its EC_50_ (1.5 nM, 0.1% DMSO) before addition of the compound for testing. A 50 μL aliquot of culture from each well was lysed in 200 μL of Z-buffer and 8 μL of chloroform, and a 100 μL aliquot of this lysate was incubated with 25 μL of 4 mg/mL ONPG for 20 min at 30 °C before reading absorption at 420 nm and 550 nm using a Biotek Synergy 2 plate reader. Non-linear regression curve fits were generated using GraphPad Prism software (version 6) using variable slope (four parameter) dose–response analysis.

## Supporting Information

File 1 Compound library structures and screening results.

File 2 Full dose–response curves.

File 3 Competition assay efficacy and potency data.
