# Supplementary material for: Non-native autoinducer analogs capable of modulating the SdiA quorum sensing receptor in Salmonella enterica serovar Typhimurium
Source: Beilstein J Org Chem. 2018 Oct 17;14:2651–64. doi: 10.3762/bjoc.14.243 (PMC6204753; doi:10.3762/bjoc.14.243)
Supplement: File 1 — Compound library structures and screening results. [file Beilstein_J_Org_Chem-14-2651-s001.pdf]

Supporting Information File 1

for

Non-native autoinducer analogs capable of modulating the SdiA quorum sensing receptor in *Salmonella enterica* serovar Typhimurium

Matthew J. Styles and Helen E. Blackwell*

Address: Department of Chemistry, University of Wisconsin–Madison, 1101 University Avenue, Madison, WI 53706, USA

* Corresponding author

Email: Helen E. Blackwell - blackwell@chem.wisc.edu

Compound library structures and screening results

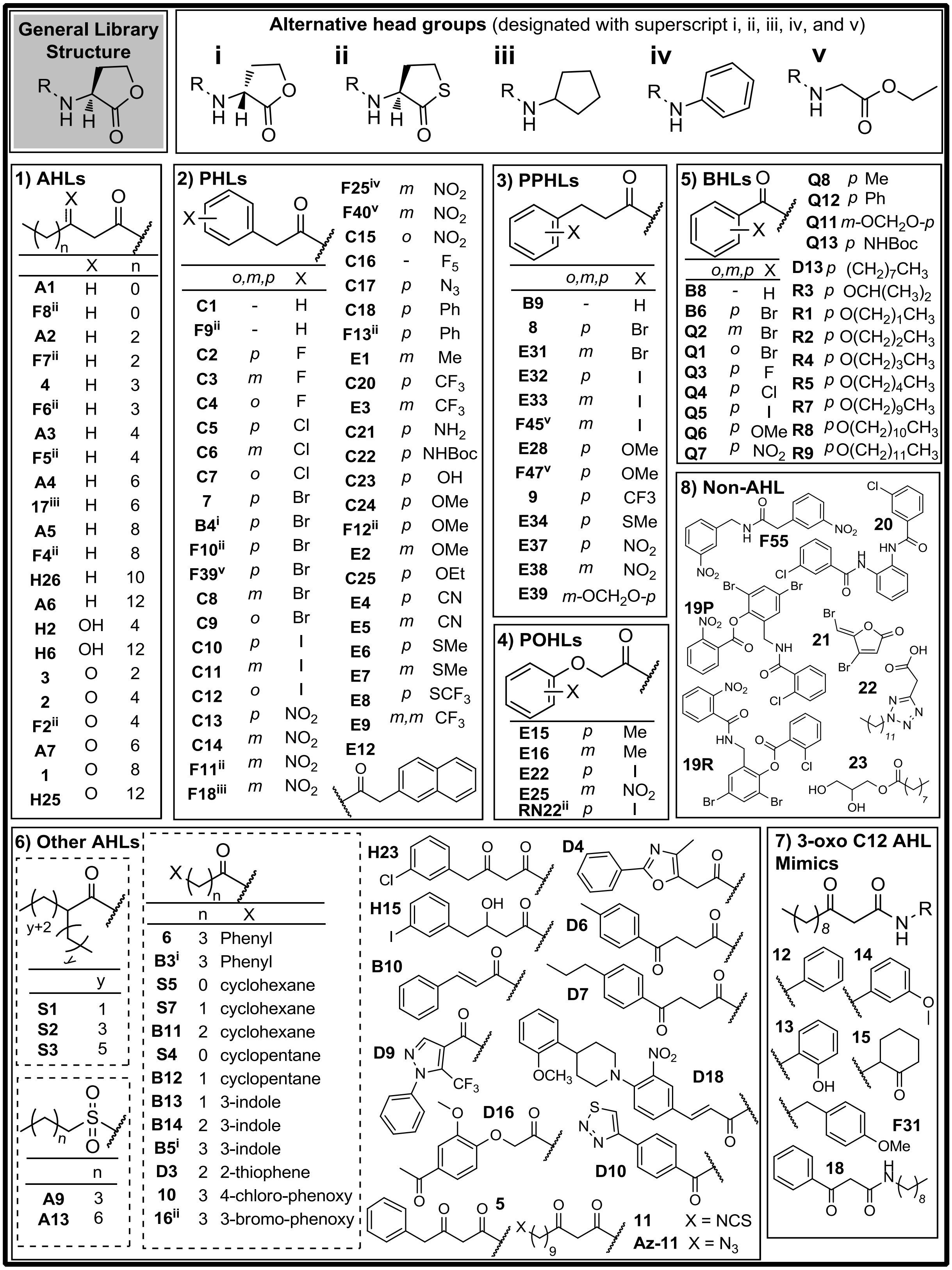

Figure S1: Structures of compounds evaluated in this study. Compounds listed by general structure class. Original sub-library letter names (A, B, C, D, E, F, H, Q, R, S, and **1–22**) from our prior publications are indicated in compound names. See main text.

Table S1: Single-concentration screening data in *S.* Typhimurium-pJNS25 SdiA reporter strain.[a]

	Agonism[b]		Antagonism[c]	
**compound**	**1 μM**	**100 μM**	**1 μM**	**100 μM**
	Activity (%)[d]	Activity (%)[d]	Inhibition (%)[e]	Inhibition (%)[e]
**A1**	41 ± 6	104 ± 8	7 ± 5	-10 ± 11
**F8**	81 ± 1	127 ± 11	-5 ± 10	-23 ± 11
**A2**	84 ± 5	83 ± 2	15 ± 4	3 ± 1
**F7**	78 ± 5	117 ± 9	7 ± 9	-12 ± 4
**4**	66 ± 9	59 ± 9	26 ± 4	16 ± 5
**F6**	78 ± 1	87 ± 9	5 ± 5	-2 ± 7
**A3**	76 ± 7	89 ± 13	10 ± 8	13 ± 14
**F5**	85 ± 2	79 ± 4	3 ± 6	0 ± 6
**A4**	59 ± 3	75 ± 11	12 ± 19	18 ± 7
**17**	3 ± 4	47 ± 3	26 ± 12	28 ± 5
**A5**	45 ± 2	57 ± 5	17 ± 10	43 ± 4
**F4**	62 ± 2	93 ± 16	-2 ± 11	2 ± 5
**H26**	19 ± 6	39 ± 12	5 ± 14	0 ± 8
**A6**	12 ± 3	39 ± 3	17 ± 12	38 ± 5
**H2**	77 ± 4	74 ± 12	8 ± 5	12 ± 16
**H6**	3 ± 3	51 ± 14	15 ± 1	15 ± 13
**3**	75 ± 14	75 ± 15	6 ± 16	20 ± 2
**2**	78 ± 11	86 ± 18	-2 ± 9	9 ± 5
**F2**	116 ± 6	88 ± 19	-19 ± 12	-19 ± 15
**A7**	86 ± 9	100 ± 4	-4 ± 19	-2 ± 10
**1**	71 ± 8	94 ± 8	2 ± 17	15 ± 16
**H25**	55 ± 8	57 ± 10	1 ± 13	-9 ± 15
**C1**	40 ± 4	90 ± 5	9 ± 12	-6 ± 5
**F9**	76 ± 1	113 ± 6	-1 ± 4	-13 ± 1
**C2**	56 ± 1	93 ± 5	2 ± 8	-8 ± 18
**C3**	56 ± 2	111 ± 9	-1 ± 15	-18 ± 12
**C4**	45 ± 3	121 ± 14	3 ± 6	-31 ± 10
**C5**	65 ± 8	85 ± 4	13 ± 6	18 ± 7
**C6**	85 ± 4	120 ± 2	9 ± 16	-20 ± 6
**C7**	75 ± 10	132 ± 6	22 ± 6	-32 ± 5
**7**	71 ± 10	68 ± 11	26 ± 9	21 ± 10
**B4**	26 ± 6	65 ± 3	41 ± 15	16 ± 11
**F10**	68 ± 3	57 ± 8	8 ± 12	-3 ± 7

[a]Compounds listed according to order in Figure S1. [b]Agonism and [c]antagonism assays performed as described in the Experimental Section at 1 μM and 100 μM compound. All assays performed in triplicate. [d]Activation reported as the average (±STD) of the percentage (%) of activity normalized to 10 μM **2** (OOHL). [e]Inhibition reported as the average (±STD) of the change in the activity (%) from 10 nM **2** (OOHL). [f]Cell growth inhibited at this compound concentration.

Continued…	**Agonism**[b]		**Antagonism**[c]	
**compound**	**1 μM**	**100 μM**	**1 μM**	**100 μM**
	Activity (%)[d]	Activity (%)[d]	Inhibition (%)[e]	Inhibition (%)[e]
**F39**	16.4 ± 0.4	31 ± 3	27 ± 9	53 ± 11
**C8**	88 ± 10	100 ± 15	21 ± 10	-22 ± 9
**C9**	97 ± 13	156 ± 10	9 ± 8	-48 ± 13
**C10**	83 ± 1	95 ± 15	28 ± 6	27 ± 13
**C11**	87 ± 7	109 ± 7	15 ± 11	-4 ± 2
**C12**	72 ± 2	125 ± 13	9 ± 13	-24 ± 12
**C13**	58 ± 4	98 ± 4	24 ± 4	14 ± 9
**C14**	79 ± 8	110 ± 9	0 ± 3	-17 ± 9
**F11**	87 ± 6	103 ± 17	-11 ± 7	-16 ± 13
**F18**	61 ± 6	69 ± 13	4 ± 6	15 ± 3
**F25**	14 ± 2	74 ± 9	20 ± 4	-2 ± 14
**F40**	16 ± 3	50 ± 8	21 ± 8	43 ± 4
**C15**	22 ± 2	110 ± 10	5 ± 2	-17 ± 18
**C16**	26 ± 2	141 ± 9	5 ± 3	-32 ± 18
**C17**	59 ± 2	103 ± 15	13 ± 11	9 ± 6
**C18**	28 ± 1	48 ± 13	11 ± 3	52 ± 10
**F13**	43 ± 6	44 ± 13	23 ± 4	46 ± 15
**E1**	84 ± 3	97 ± 15	-6 ± 7	-14 ± 13
**C20**	69 ± 16	86 ± 6	15 ± 10	8 ± 6
**E3**	75 ± 2	89 ± 14	-14 ± 16	5 ± 8
**C21**	17 ± 4	76 ± 7	4 ± 12	13 ± 4
**C22**	16 ± 3	56 ± 5	-9 ± 2	6 ± 6
**C23**	65 ± 11	90 ± 8	6 ± 2	16 ± 3
**C24**	75 ± 10	87 ± 2	-11 ± 3	4 ± 16
**F12**	93 ± 9	86 ± 10	-13 ± 10	-21 ± 8
**E2**	73 ± 4	93 ± 14	-5 ± 1	-8 ± 14
**C25**	64 ± 11	92 ± 1	12 ± 8	7 ± 5
**E4**	74 ± 1	89 ± 16	12 ± 6	-17 ± 18
**E5**	90 ± 12	100 ± 16	18 ± 8	-8 ± 4
**E6**	61 ± 6	64 ± 16	25 ± 16	44 ± 13
**E7**	81 ± 18	93 ± 15	20 ± 14	12 ± 13
**E8**	52 ± 10	82 ± 10	34 ± 11	31 ± 7
**E9**	42 ± 15	61 ± 1	18 ± 8	21 ± 8
**E12**	77 ± 16	67 ± 5	-13 ± 9	0 ± 15
**B9**	55 ± 3	96 ± 5	13 ± 10	18 ± 14
**8**	77 ± 5	87 ± 16	15 ± 8	4 ± 3
**E31**	70 ± 18	56 ± 8	13 ± 10	6 ± 6
**E32**	36 ± 1	83 ± 7	19 ± 14	24 ± 13
**E33**	34 ± 5	51 ± 4	20 ± 6	38 ± 10
**F45**	4.8 ± 0.1	13 ± 2	8 ± 10	92 ± 2

Continued…	**Agonism**[b]		**Antagonism**[c]	
**compound**	**1 μM**	**100 μM**	**1 μM**	**100 μM**
	Activity (%)[d]	Activity (%)[d]	Inhibition (%)[e]	Inhibition (%)[e]
**E28**	71 ± 12	66 ± 7	11 ± 18	6 ± 15
**F47**	4 ± 2	50 ± 5	3 ± 14	36 ± 10
**9**	52 ± 4	59 ± 6	32 ± 10	11 ± 16
**E34**	33 ± 4	53 ± 5	21 ± 15	39 ± 8
**E37**	57 ± 4	70 ± 15	34 ± 5	23 ± 5
**E38**	35 ± 2	56 ± 9	15 ± 11	32 ± 6
**E39**	41 ± 6	72 ± 12	10 ± 15	12 ± 3
**E15**	86 ± 4	126 ± 3	-3 ± 5	-17 ± 7
**E16**	86 ± 6	104 ± 4	-2 ± 8	-4 ± 5
**E22**	96 ± 6	136 ± 6	-5 ± 11	-15 ± 8
**E25**	78 ± 9	76 ± 7	5 ± 18	9 ± 12
**RN22**	94 ± 19	76 ± 5	-4 ± 9	3 ± 9
**B8**	12 ± 1	77 ± 4	26 ± 3	-13 ± 11
**B6**	12 ± 3	51 ± 1	25 ± 11	-14 ± 17
**Q2**	10 ± 4	72 ± 1	17 ± 2	9 ± 13
**Q1**	8 ± 2	74 ± 3	2 ± 5	-3 ± 18
**Q3**	3 ± 5	55 ± 3	34 ± 2	-26 ± 17
**Q4**	1 ± 4	26 ± 3	30 ± 4	-25 ± 15
**Q5**	0 ± 1	24 ± 4	28 ± 10	-14 ± 10
**Q6**	3 ± 2	53 ± 2	26 ± 18	-6 ± 14
**Q7**	1 ± 2	55 ± 3	28 ± 7	20 ± 9
**Q8**	2 ± 3	53 ± 5	17 ± 3	11 ± 3
**Q12**	11 ± 7	12 ± 4	20 ± 9	26 ± 14
**Q11**	16 ± 7	59 ± 12	7 ± 7	-2 ± 12
**Q13**	13 ± 6	85 ± 10	19 ± 8	-14 ± 2
**D13**	7 ± 3	6 ± 2	17 ± 7	23 ± 7
**R3**	11 ± 3	55 ± 2	23 ± 8	11 ± 10
**R1**	7 ± 1	10 ± 1	22 ± 6	13 ± 8
**R2**	7 ± 2	31 ± 8	16 ± 9	11 ± 6
**R4**	9 ± 4	13 ± 1	23 ± 9	11 ± 12
**R5**	8 ± 1	29 ± 1	10 ± 18	-1 ± 9
**R7**	6 ± 1	0 ± 2	8 ± 6	43 ± 15
**R8**	8 ± 1	23 ± 2	18 ± 5	67 ± 5
**R9**	13 ± 5	10 ± 4	22 ± 10	42 ± 13
**S1**	74 ± 6	100 ± 7	24 ± 11	-8 ± 7
**S2**	10 ± 4	57 ± 3	48 ± 3	2 ± 11
**S3**	48 ± 11	85 ± 3	25 ± 8	7 ± 13
**A9**	36 ± 1	83 ± 8	10 ± 9	13 ± 2
**A13**	16 ± 2	77 ± 11	26 ± 10	-2 ± 5
**6**	52 ± 13	65 ± 16	31 ± 6	21 ± 10

Continued…	**Agonism**[b]		**Antagonism**[c]	
**compound**	**1 μM**	**100 μM**	**1 μM**	**100 μM**
	Activity (%)[d]	Activity (%)[d]	Inhibition (%)[e]	Inhibition (%)[e]
**B3**	24 ± 3	78 ± 3	34 ± 5	16 ± 3
**S5**	-2 ± 1	69 ± 3	19 ± 4	13 ± 10
**S7**	84 ± 2	97 ± 5	7 ± 14	1 ± 7
**B11**	88 ± 15	108 ± 6	6 ± 5	8 ± 14
**S4**	59 ± 6	115 ± 7	27 ± 11	-8 ± 11
**B12**	86 ± 12	117 ± 10	7 ± 7	14 ± 9
**B13**	67 ± 13	107 ± 6	8 ± 10	13 ± 13
**B14**	41 ± 8	92 ± 5	10 ± 2	18 ± 5
**B5**	12 ± 3	57 ± 1	30 ± 15	-8 ± 5
**D3**	58 ± 1	82 ± 4	10 ± 2	6 ± 9
**10**	30 ± 4	52 ± 11	9 ± 12	5 ± 15
**16**	50 ± 7	19 ± 2	8 ± 14	84 ± 6
**5**	67 ± 17	69 ± 15	23 ± 9	17 ± 3
**H15**	29 ± 3	59 ± 14	6 ± 14	19 ± 11
**H23**	70 ± 8	81 ± 15	12 ± 8	1 ± 9
**B10**	12 ± 2	73 ± 5	16 ± 5	-56 ± 11
**D4**	23 ± 1	76 ± 4	28 ± 13	-6 ± 4
**D6**	41 ± 5	80 ± 5	21 ± 19	3 ± 12
**D7**	31 ± 8	74 ± 4	20 ± 6	11 ± 9
**D9**	13 ± 1	52 ± 6	26 ± 6	9 ± 1
**D10**	6 ± 2	22 ± 2	23 ± 6	14 ± 12
**D16**	13 ± 4	83 ± 4	19 ± 6	3 ± 6
**D18**	16 ± 4	53 ± 2	18 ± 8	32 ± 5
**11**	-8 ± 3	-33 ± 2	64 ± 6	128 ± 1
**Az-11**	109 ± 15	104 ± 4	-1 ± 17	-35 ± 6
**12**	-2 ± 2	16 ± 2	20 ± 19	43 ± 13
**13**	1 ± 3	61 ± 6	10 ± 8	37 ± 5
**14**	7 ± 12	61 ± 4	22 ± 19	12 ± 8
**15**	-1 ± 2	13 ± 4	16 ± 16	13 ± 8
**F31**	5 ± 2	32 ± 2	31 ± 5	15 ± 4
**18**	-1 ± 3	43 ± 3	39 ± 11	45 ± 5
**F55**	2 ± 1	37 ± 6	11 ± 4	26 ± 7
**19R**	-1 ± 4	35 ± 8	43 ± 10	38 ± 11
**19P**	-2 ± 3	-4 ± 1	39 ± 16	25 ± 16
**20**	2 ± 6	34 ± 11	35 ± 18	39 ± 17
**21**[f]	-2 ± 3	--[f]	29 ± 13	--[f]
**22**	-2 ± 3	8 ± 6	35 ± 11	23 ± 18
**23**	-1 ± 4	-2 ± 2	8 ± 14	12 ± 4
