# Supplementary material for: Non-native autoinducer analogs capable of modulating the SdiA quorum sensing receptor in Salmonella enterica serovar Typhimurium
Source: Beilstein J Org Chem. 2018 Oct 17;14:2651–64. doi: 10.3762/bjoc.14.243 (PMC6204753; doi:10.3762/bjoc.14.243)
Supplement: File 2 — Full dose–response curves. [file Beilstein_J_Org_Chem-14-2651-s002.pdf]

Supporting Information File 2

for

Non-native autoinducer analogs capable of modulating the SdiA quorum sensing receptor in *Salmonella enterica* serovar Typhimurium

Matthew J. Styles and Helen E. Blackwell*

Address: Department of Chemistry, University of Wisconsin–Madison, 1101 University Avenue, Madison, WI 53706, USA

* Corresponding author

Email: Helen E. Blackwell - blackwell@chem.wisc.edu

Full dose–response curves

Agonism and antagonism dose-response curves in support of data listed in Tables 1–4 in the main text. Plots corresponding to these data as listed on the pages below:

- **Table S1:** Pages S2–S4
  Agonism dose-response curves for compounds **F2** to **11-Az** in the *S.* Typhimurium-pJNS25 reporter strain.

- **Table S2:** Page S5
  Agonism dose-response curves for compounds **A1** to **C24** in the *S.* Typhimurium-pJNS25 reporter strain.

- **Table S3:** Pages S6–S8
  Agonism (black) and antagonism (red) dose-response curves for compounds **11 (ITC-12)** to **S2** in the *S.* Typhimurium-pJNS25 reporter strain. Antagonists competed against the EC_{90} of **2** (OOHL), 10 nM.

- **Table S4:** Page S9
  Antagonism dose-response curves for compounds **11 (ITC-12)**, **R8**, **16**, and **F45** in the *E. coli* JLD271-pJN105SE-pSC11SE reporter strain. Antagonists competed against the EC_{50} of **2** (OOHL), 1.5 nM. Agonism dose-response curve for **2** (OOHL) also included.

All assay protocols are described in the Experimental Section in the main text.

F2

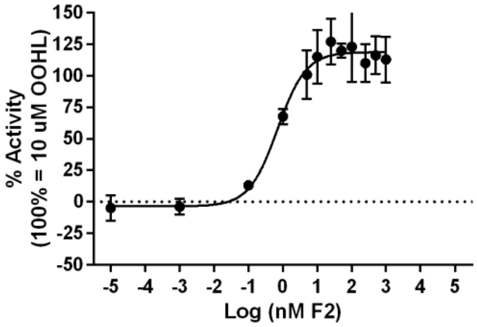

2 (OOHL)

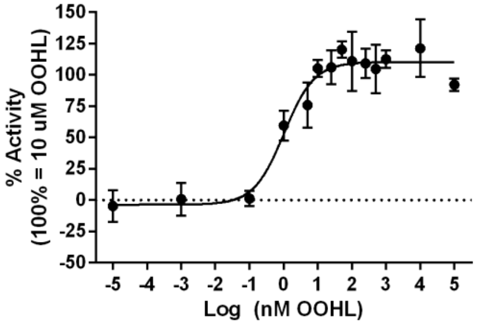

F5

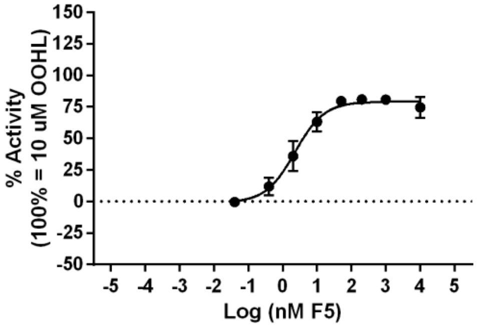

F11

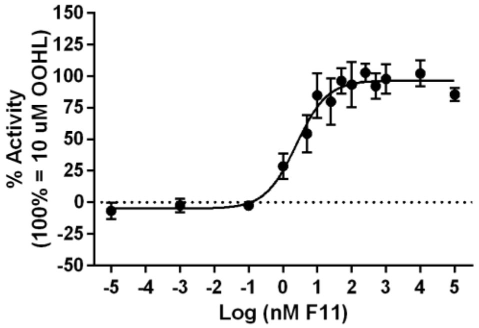

RN22

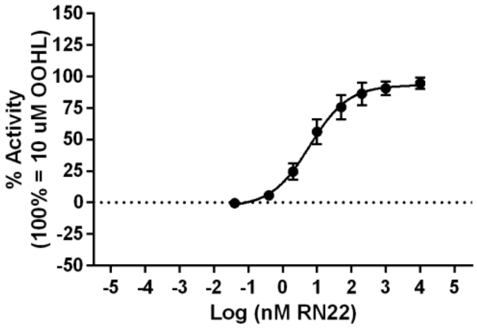

E22

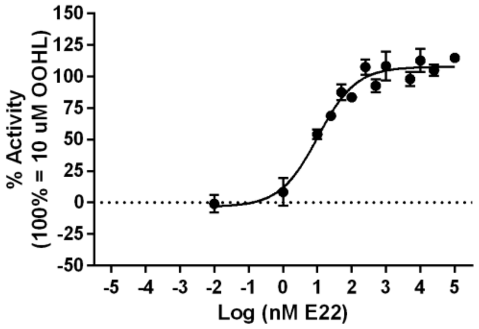

C8

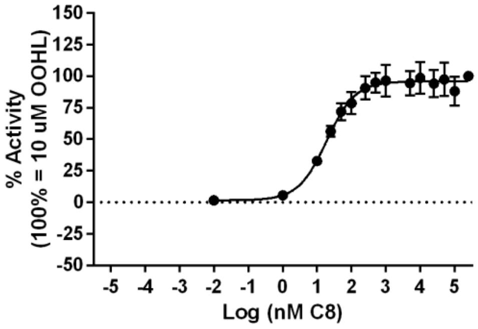

C11

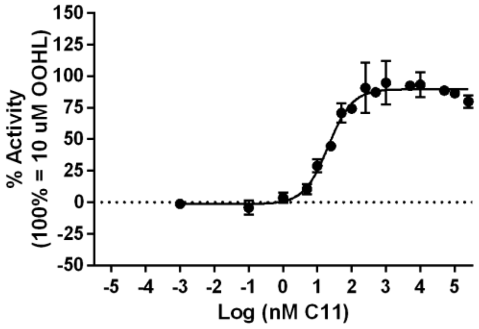

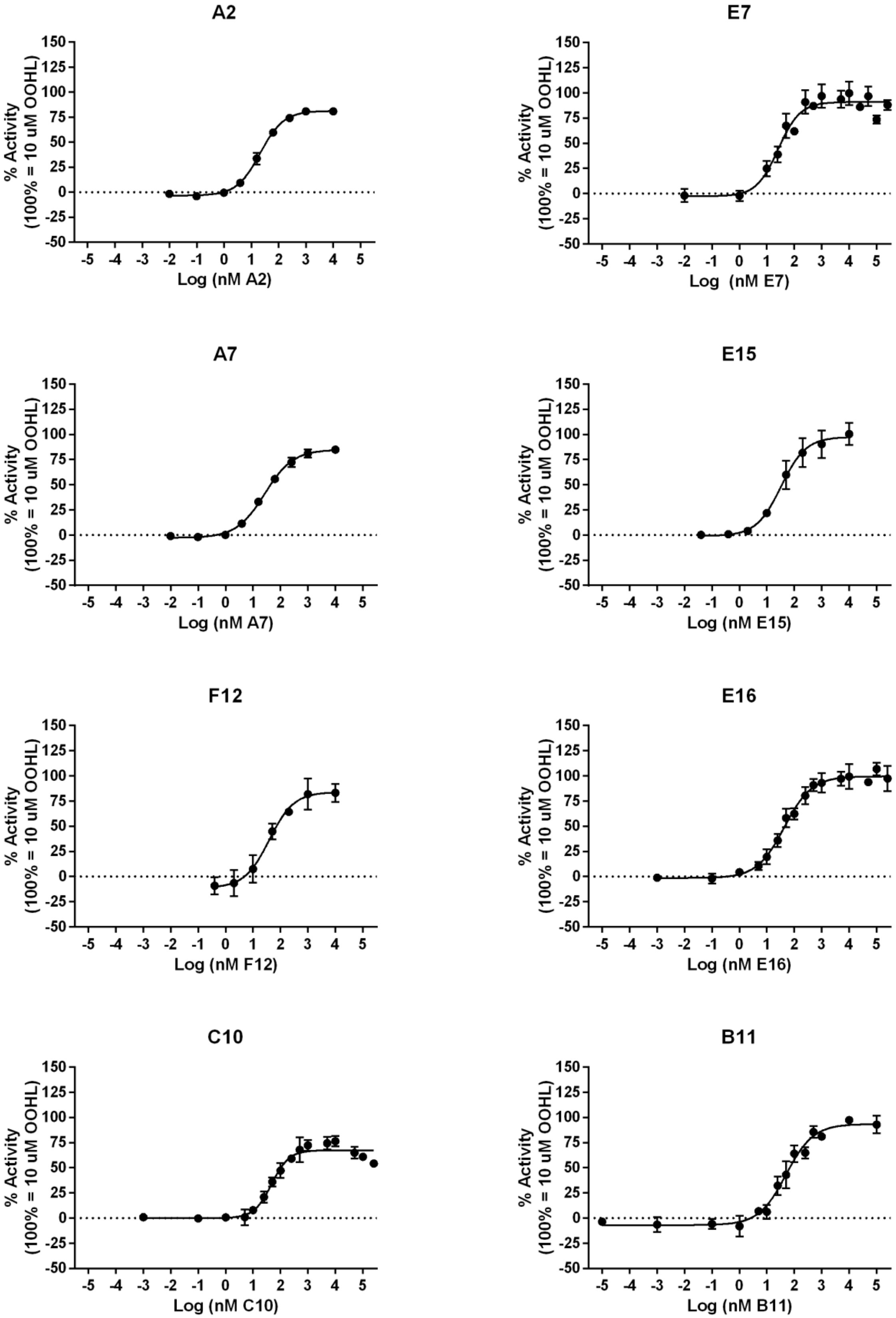
A2
% Activity (100% = 10 uM OOHL)
Log (nM A2)
E7
% Activity (100% = 10 uM OOHL)
Log (nM E7)
A7
% Activity (100% = 10 uM OOHL)
Log (nM A7)
E15
% Activity (100% = 10 uM OOHL)
Log (nM E15)
F12
% Activity (100% = 10 uM OOHL)
Log (nM F12)
E16
% Activity (100% = 10 uM OOHL)
Log (nM E16)
C10
% Activity (100% = 10 uM OOHL)
Log (nM C10)
B11
% Activity (100% = 10 uM OOHL)
Log (nM B11)

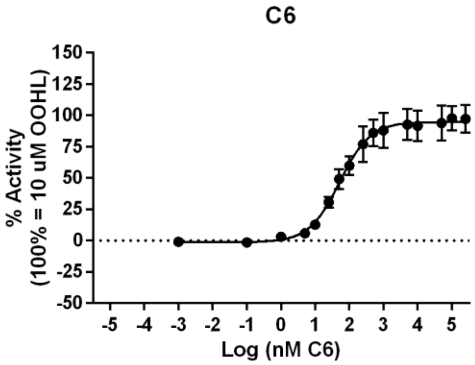
C6
% Activity
(100% = 10 uM OOHL)
150 125 100 75 50 25 0 -25 -50
-5 -4 -3 -2 -1 0 1 2 3 4 5
Log (nM C6)

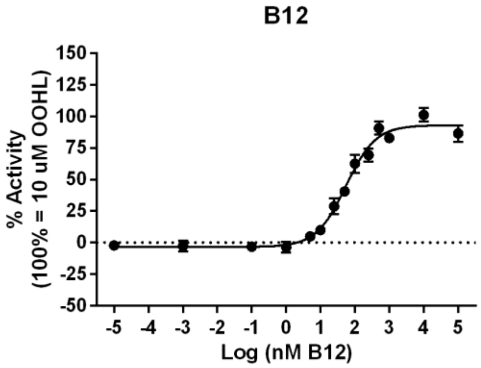
B12
% Activity
(100% = 10 uM OOHL)
150 125 100 75 50 25 0 -25 -50
-5 -4 -3 -2 -1 0 1 2 3 4 5
Log (nM B12)

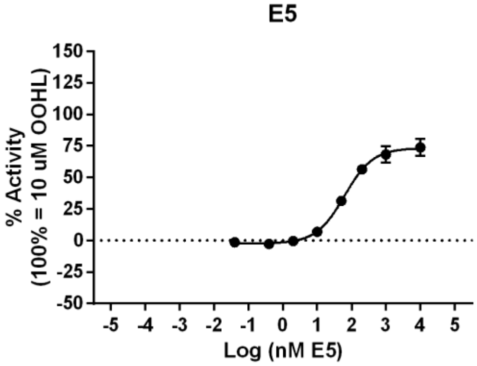
E5
% Activity
(100% = 10 uM OOHL)
150 125 100 75 50 25 0 -25 -50
-5 -4 -3 -2 -1 0 1 2 3 4 5
Log (nM E5)

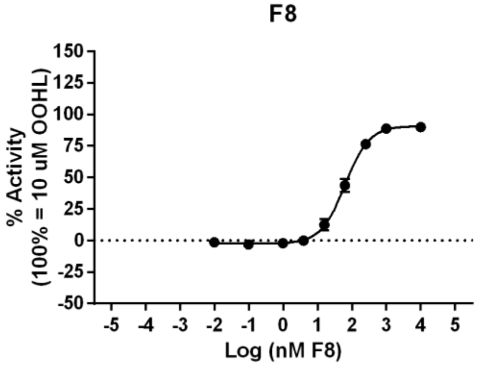
F8
% Activity
(100% = 10 uM OOHL)
150 125 100 75 50 25 0 -25 -50
-5 -4 -3 -2 -1 0 1 2 3 4 5
Log (nM F8)

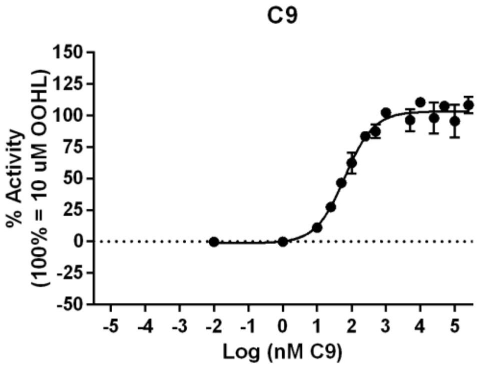
C9
% Activity
(100% = 10 uM OOHL)
150 125 100 75 50 25 0 -25 -50
-5 -4 -3 -2 -1 0 1 2 3 4 5
Log (nM C9)

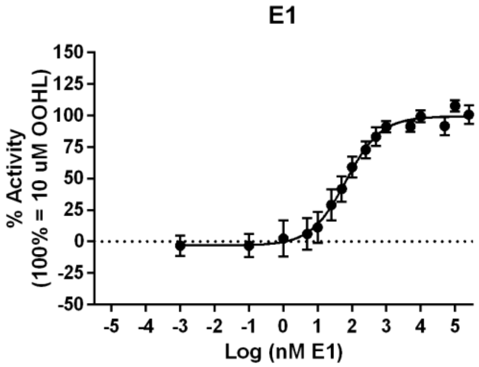
E1
% Activity
(100% = 10 uM OOHL)
150 125 100 75 50 25 0 -25 -50
-5 -4 -3 -2 -1 0 1 2 3 4 5
Log (nM E1)

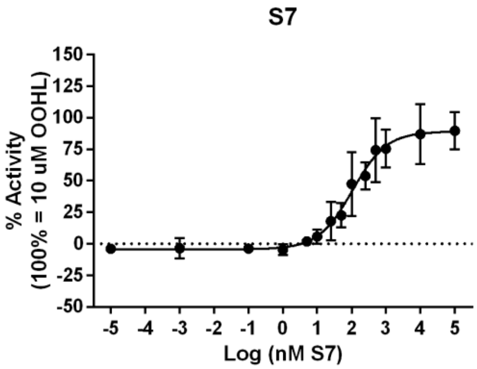
S7
% Activity
(100% = 10 uM OOHL)
150 125 100 75 50 25 0 -25 -50
-5 -4 -3 -2 -1 0 1 2 3 4 5
Log (nM S7)

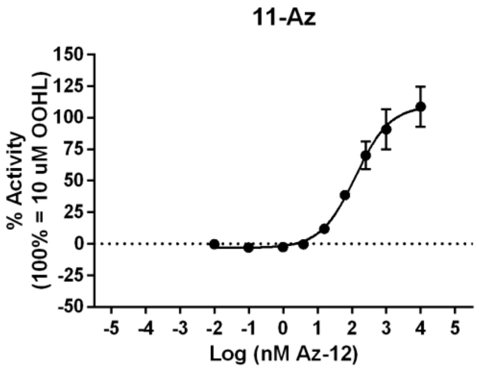
11-Az
% Activity
(100% = 10 uM OOHL)
150 125 100 75 50 25 0 -25 -50
-5 -4 -3 -2 -1 0 1 2 3 4 5
Log (nM Az-12)

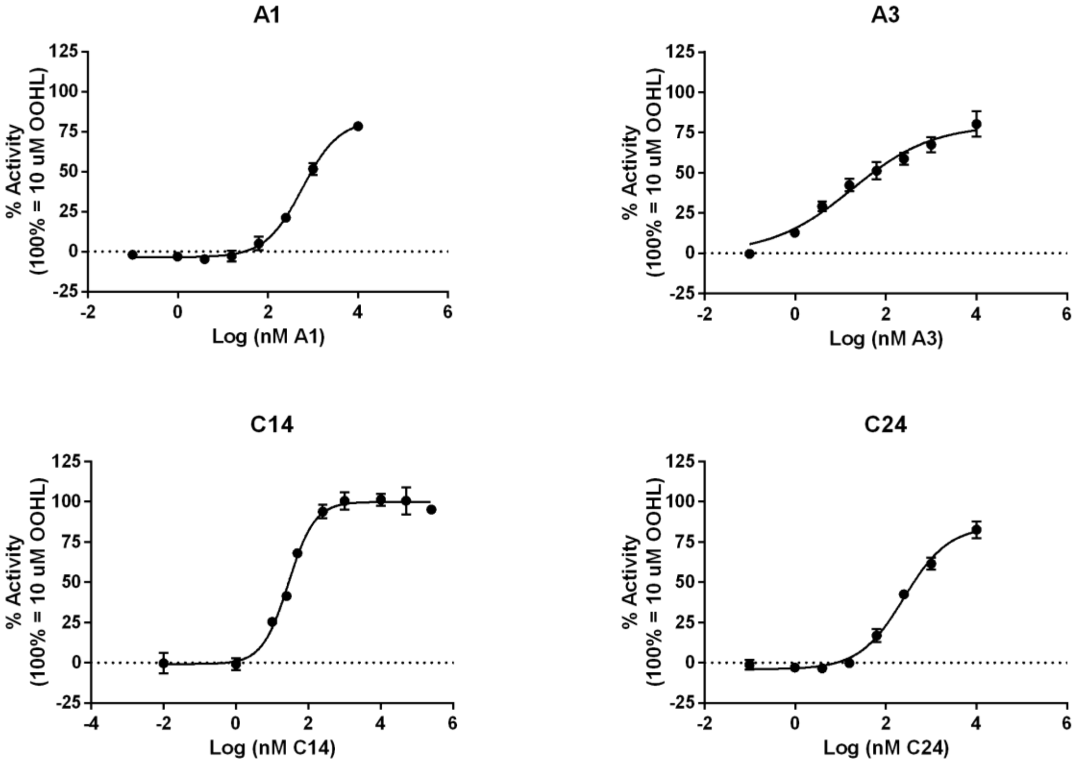
A1
% Activity (100% = 10 uM OOHL)
Log (nM A1)
A3
% Activity (100% = 10 uM OOHL)
Log (nM A3)
C14
% Activity (100% = 10 uM OOHL)
Log (nM C14)
C24
% Activity (100% = 10 uM OOHL)
Log (nM C24)

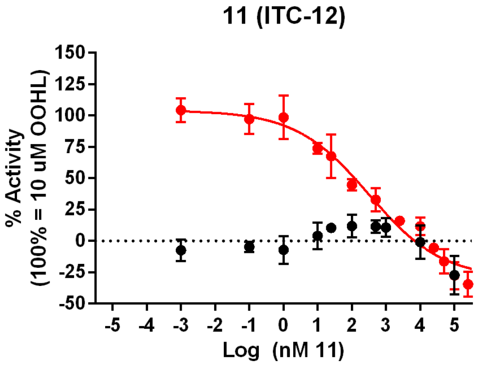
11 (ITC-12)
% Activity
(100% = 10 uM OOHL)
Log (nM 11)

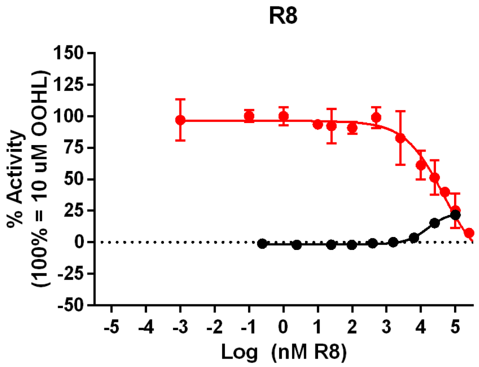
R8
% Activity
(100% = 10 uM OOHL)
Log (nM R8)

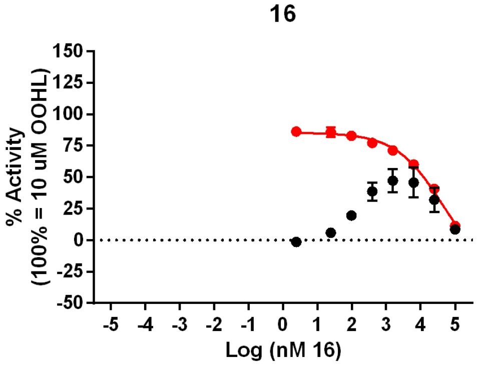
16
% Activity
(100% = 10 uM OOHL)
Log (nM 16)

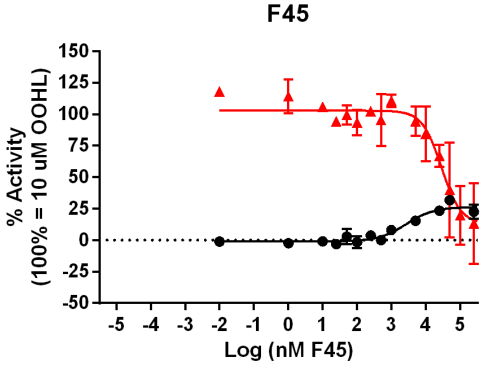
F45
% Activity
(100% = 10 uM OOHL)
Log (nM F45)

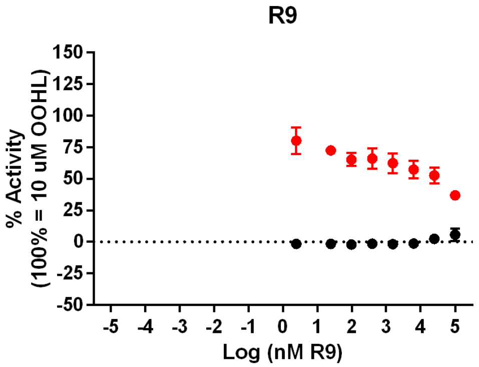
R9
% Activity
(100% = 10 uM OOHL)
Log (nM R9)

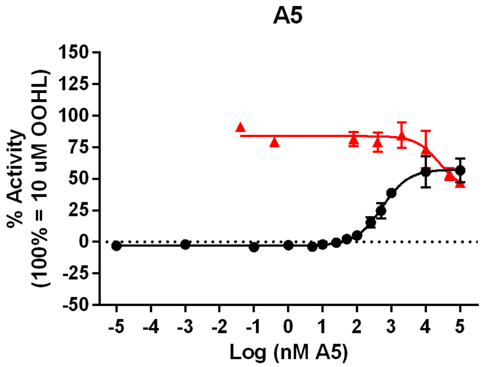
A5
% Activity
(100% = 10 uM OOHL)
Log (nM A5)

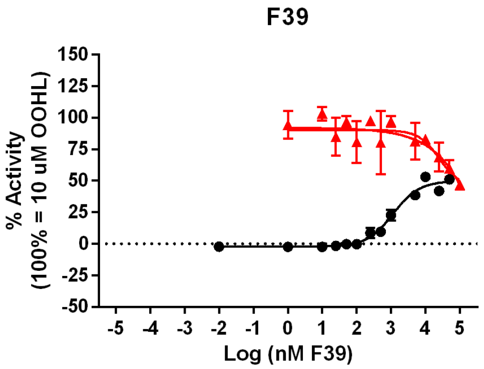
F39
% Activity
(100% = 10 uM OOHL)
Log (nM F39)

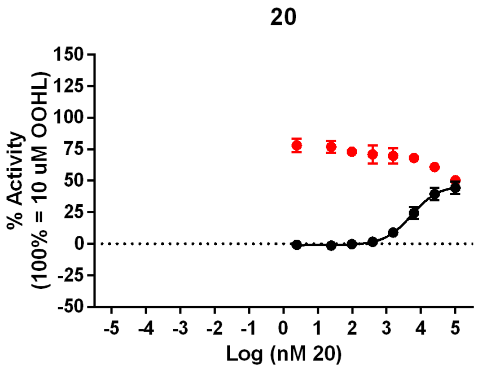
20
% Activity
(100% = 10 uM OOHL)
Log (nM 20)

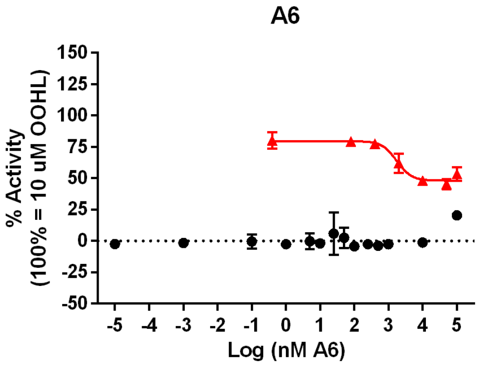
A6
% Activity
(100% = 10 uM OOHL)
Log (nM A6)

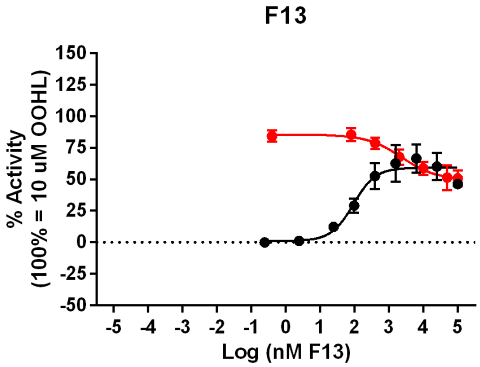
F13
% Activity
(100% = 10 uM OOHL)
Log (nM F13)

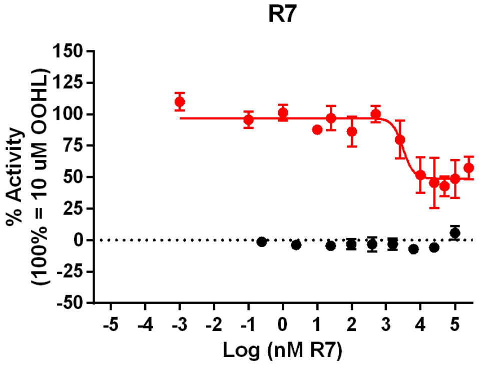
R7
% Activity
(100% = 10 uM OOHL)
Log (nM R7)

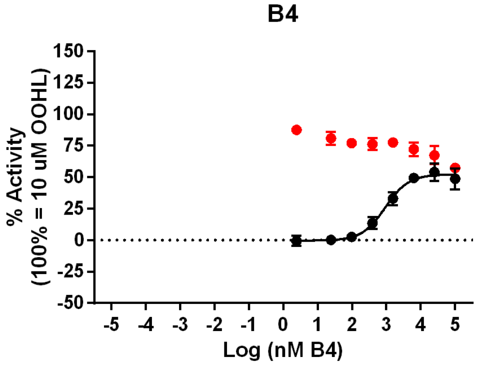
B4
% Activity
(100% = 10 uM OOHL)
Log (nM B4)

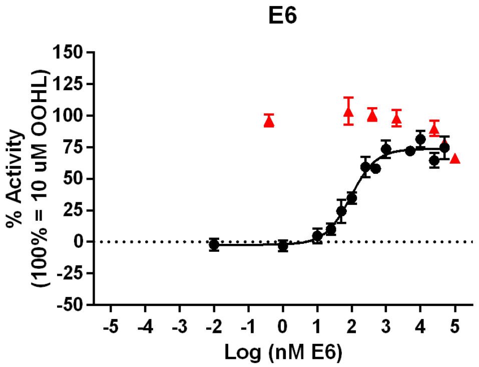
E6
% Activity
(100% = 10 uM OOHL)
Log (nM E6)

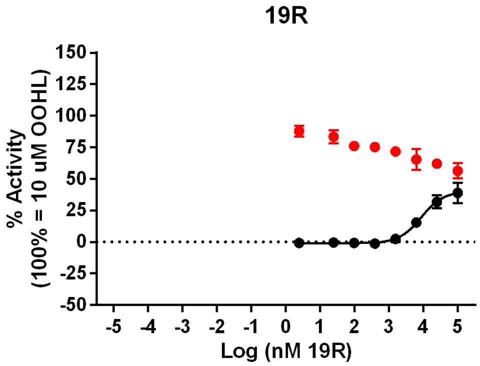
19R
% Activity
(100% = 10 uM OOHL)
Log (nM 19R)

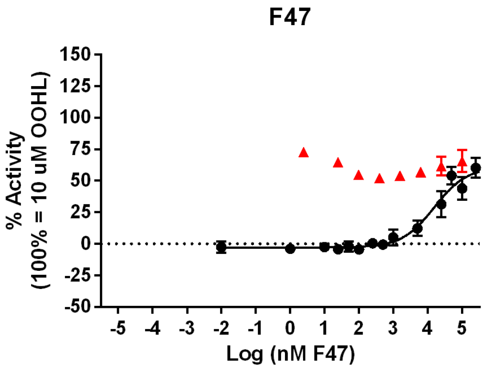
F47
% Activity
(100% = 10 uM OOHL)
Log (nM F47)

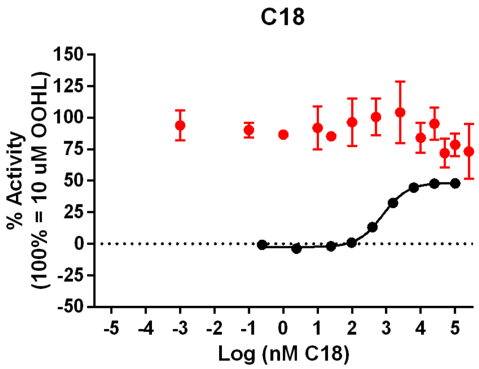
C18
% Activity
(100% = 10 uM OOHL)
Log (nM C18)

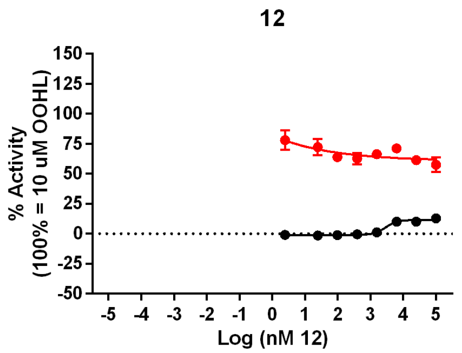
12
% Activity
(100% = 10 uM OOHL)
150
125
100
75
50
25
0
-25
-50
-5 -4 -3 -2 -1 0 1 2 3 4 5
Log (nM 12)

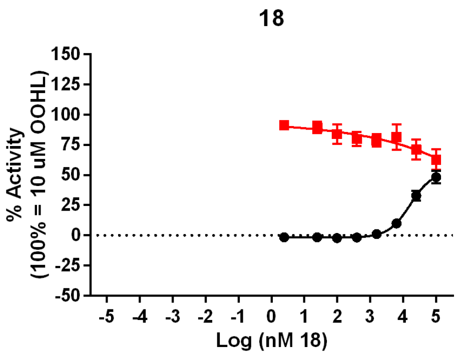
18
% Activity
(100% = 10 uM OOHL)
150
125
100
75
50
25
0
-25
-50
-5 -4 -3 -2 -1 0 1 2 3 4 5
Log (nM 18)

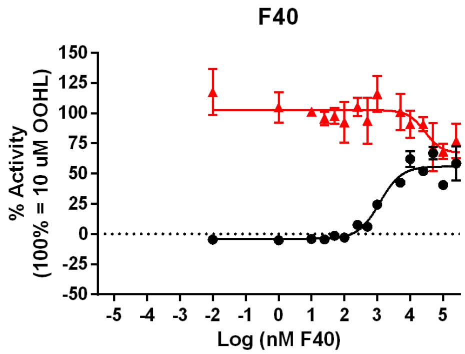
F40
% Activity
(100% = 10 uM OOHL)
150
125
100
75
50
25
0
-25
-50
-5 -4 -3 -2 -1 0 1 2 3 4 5
Log (nM F40)

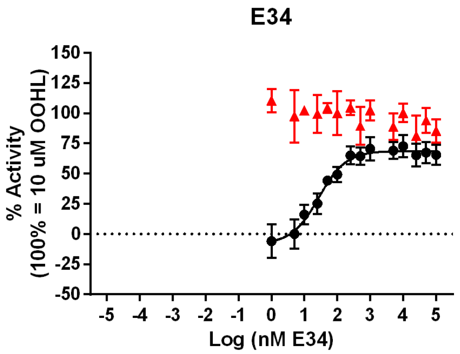
E34
% Activity
(100% = 10 uM OOHL)
150
125
100
75
50
25
0
-25
-50
-5 -4 -3 -2 -1 0 1 2 3 4 5
Log (nM E34)

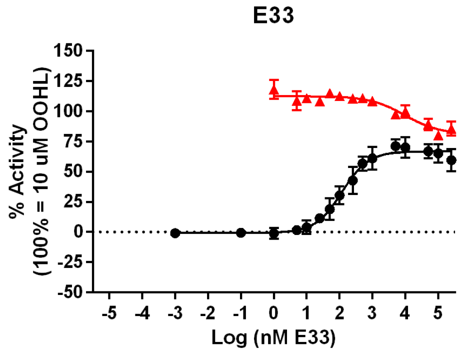
E33
% Activity
(100% = 10 uM OOHL)
150
125
100
75
50
25
0
-25
-50
-5 -4 -3 -2 -1 0 1 2 3 4 5
Log (nM E33)

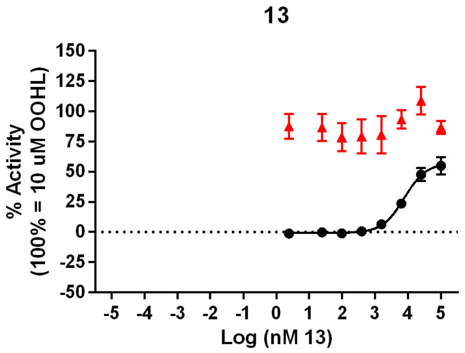
13
% Activity
(100% = 10 uM OOHL)
150
125
100
75
50
25
0
-25
-50
-5 -4 -3 -2 -1 0 1 2 3 4 5
Log (nM 13)

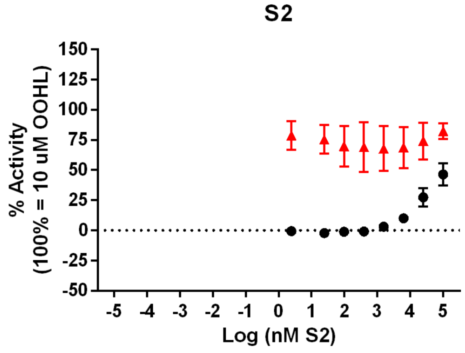
S2
% Activity
(100% = 10 uM OOHL)
150
125
100
75
50
25
0
-25
-50
-5 -4 -3 -2 -1 0 1 2 3 4 5
Log (nM S2)

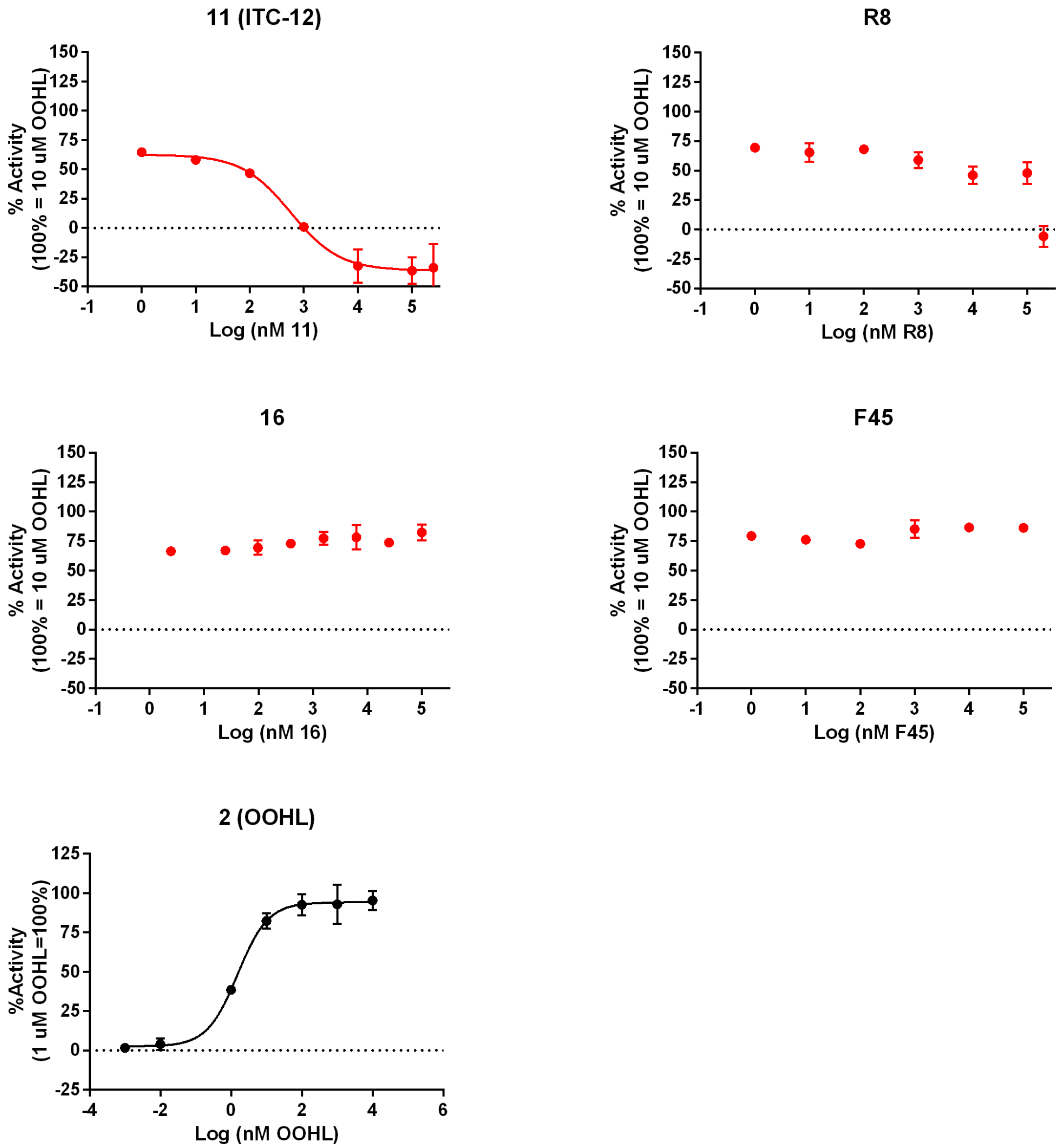
11 (ITC-12)
% Activity (100% = 10 uM OOHL)
Log (nM 11)
R8
% Activity (100% = 10 uM OOHL)
Log (nM R8)
16
% Activity (100% = 10 uM OOHL)
Log (nM 16)
F45
% Activity (100% = 10 uM OOHL)
Log (nM F45)
2 (OOHL)
%Activity (1 uM OOHL=100%)
Log (nM OOHL)
