# Supplementary material for: Non-native autoinducer analogs capable of modulating the SdiA quorum sensing receptor in Salmonella enterica serovar Typhimurium
Source: Beilstein J Org Chem. 2018 Oct 17;14:2651–64. doi: 10.3762/bjoc.14.243 (PMC6204753; doi:10.3762/bjoc.14.243)
Supplement: File 3 — Competition assay efficacy and potency data. [file Beilstein_J_Org_Chem-14-2651-s003.pdf]

Supporting Information File 3

for

Non-native autoinducer analogs capable of modulating the SdiA quorum sensing receptor in *Salmonella enterica* serovar Typhimurium

Matthew J. Styles and Helen E. Blackwell*

Address: Department of Chemistry, University of Wisconsin–Madison, 1101 University Avenue, Madison, WI 53706, USA

* Corresponding author

Email: Helen E. Blackwell - blackwell@chem.wisc.edu

Competition assay efficacy and potency data

Table S1: Efficacy and potency data for OOHL (**2**) in competition against antagonists **F45**, **R8**, and **11** at varying concentrations.[a] See Figure 5 in main article for dose–response curves.

**Compound**	**Concentration** (μM)	**Activation** (%)	**95% CI** (%)[b]	**EC_{50}** (nM)	**95% CI** (nM)[c]
No antagonist	0	110[d]	103–117	1.03	0.549–1.48
**F45**	0.1	84	79–89	1.77	1.04–3.01
**F45**	1	77	73–80	1.33	0.78–2.26
**F45**	10	56	52–61	4.11	1.40–12.1
**F45**	100	21	14–28	--[e]	--
**R8**	0.1	96	91–100	1.57	1.03–2.40
**R8**	1	95	91–99	1.51	0.98–2.32
**R8**	10	114[d]	108–120	2.52	1.44–4.43
**R8**	100	79	75–83	10.6	4.8–23.5
**11**	0.1	97	92–102	72.3	48.3–108
**11**	1	97	91–103	220	151–321
**11**	10	78	73–82	1313	999–1725
**11**	100	20	15–26	4673	2510–8701

[a]All assays are averages of biological triplicates in the *S.* Typhimurium-pJNS25 SdiA reporter strain (see Experimental Section). [b]CI = confidence interval for the Activation % ($p = 0.05$). [c]CI = confidence interval for the EC_{50} ($p = 0.05$). [d]Not significantly different than the positive control ($p = 0.05$). [e]Non-linear fit could not be completed.
